# Optimal combination treatment regimens of vaccine and radiotherapy augment tumor-bearing host immunity

**DOI:** 10.1038/s42003-020-01598-6

**Published:** 2021-01-19

**Authors:** Fayun Zhang, Zifeng Zheng, Apurba Kumar Barman, Zihao Wang, Luyao Wang, Wenfeng Zeng, Luoyang Wang, Yan Qin, Asmita Pandey, Chunling Zhang, Wei Liang

**Affiliations:** 1 Protein & Peptide Pharmaceutical Laboratory, Institute of Biophysics, Chinese Academy of Sciences, Beijing, 100101 People’s Republic of China; 2 University of Chinese Academy of Sciences, Beijing, 100049 People’s Republic of China; 3 Pharmacology Laboratory, Department of Pharmacy, School of Life Science and Health, Ranada Prasad Shaha University, Naryanganj, 1400 Bangladesh; 4 Department of Chemical Engineering, Tsinghua University, Beijing, 100084 People’s Republic of China

**Keywords:** Cancer prevention, Cancer therapy

## Abstract

A major obstacle to immunotherapy is insufficient infiltration of effector immune cells into the tumor microenvironment. Radiotherapy greatly reduces tumor burden but relapses often occur. Here we show that the immunosuppressive tumor microenvironment was gradually established by recruiting Tregs after radiation. Despite tumors being controlled after depletion of Tregs in the irradiated area, improvement of mice survival remained poor. A much better antitumor effect was achieved with vaccination followed by radiation than other treatments. Vaccination followed by radiation recruited more effector T cells in tumor regions, which responded to high levels of chemokines. Sequential combination of vaccination and radiotherapy could elicit distinct host immune responses. Our study demonstrated that optimal combination of irradiation and vaccination is required to achieve effective antitumor immune responses. We propose a combination regimen that could be easily translated into the clinic and offer an opportunity for rational combination therapies design in cancer treatment.

## Abstract (web summary)

Zhang et al. use mouse tumor models to show that the optimum treatment regime for Treg-induced radiation resistance is vaccine administration followed by radiation therapy. This finding could inform the design of future combinatorial cancer therapies in the clinic.

## Introduction

The immunosuppressive microenvironment induced by primary tumors favors tumor progression^1^. Recruitment of regulatory T cells (Tregs) into the tumor microenvironment (TME) has a major immunosuppressive effect that dampens spontaneous T-cell immunity^2,3^, leading to tumor growth and poor patient outcome^4^. Clinically, high-density Tregs and exhausted CD8^+^ T cells have been found in liver cancer^5^, gastric cancer^6^, and epithelial ovarian cancer^7^, and are closely associated with tumor progression. Conversely, high ratios of CD8^+^ T cells to Tregs are positively associated with better survival in many types of cancer^7–9^. This suggests that CD8^+^/Treg ratio could be a marker correlated with cancer progression and patient survival.

Radiotherapy (RT) is currently used as a standard therapy in up to 50% of cancer patients^10^. Increasing evidences show that the potential immunosuppressive effects induced by radiation could hinder T-cell activation, which in turn prevented the irradiated tumor from converting into an “in situ” vaccine. Attempts have been made to prevent Treg activity by modulating Treg populations in tumors but failed to improve Treg-induced immunosuppression, resulting in poor immunity^11^. A recent study has shown that even apoptotic Treg cells sustain and amplify their suppressor capacity to abolish antitumor T-cell immunity^12^. Importantly, clinical data have revealed that RT alone seldom efficiently triggers antitumor immunity in cancer patients^13,14^. One major reason could be that RT alone fails to induce enough peripheral effector immune cells and recruit them into irradiated tumors. The optimal therapeutic synergy is becoming a critical issue in clinical cancer treatment.

Vaccines or immune checkpoint inhibitors that target cytotoxic T-lymphocyte-associated antigen CTLA-4 or programmed death PD-1 are reported to enhance active antitumor immune responses^15–17^. Protein antigens as vaccines have the ability to stimulate lymphocyte responses and induce an efficient CTL response^18^. However, the insufficient infiltration of antitumor effector immune cells into the solid tumor^19^ might be a major obstacle to prevent immunotherapies from exerting their full potential. Several studies have highlighted the potential of radiation to convert tumors into inflamed peripheral tissues. This is achieved by inducing chemokines like CCL5 and CXCL9/10, which are involved in the recruitment of effector T cells that express their receptors such as CCR5 and CXCR3^20,21^. Overexpression of CXCL9, CXCL10, CXCL11, and CCL5 in pretreated tumors is associated with treatment responsiveness^22^. Blockade of IFN-γ or CXCR3 can lead to a significant decrease in accumulation of CD8^+^ T cells at tumor sites^23^. In addition, sufficient T cell infiltration into the TME is a prerequisite for overcoming tumor resistance to checkpoint blockade^24^. The loss of tumor MHC class I (MHC-I) presentation is commonly found in malignant cells, which induces tumor immune escape^25^ and elicits immunotherapy resistance^26^. Although immunotherapies are considered to provide sufficient T cells for anti-tumor effects, it remains unclear what mediators are required to improve responses to immunotherapies for immune-resistant cancers.

Several studies have shown augmented immune responses when radiation is combined with tumor-specific vaccines^27,28^. Tumor vaccines are usually considered as a booster for the immune cells induced by RT, therefore, administration of vaccines after RT can achieve potent antitumor activity^27^. Other researchers have shown that vaccines followed by radiation augment antitumor immunity in the irradiated tumors^29^. However, few studies focused on which sequential combination of vaccination and RT could elicit sufficient effector T cells to overcome the functions of Tregs in advanced and metastatic tumors. The optimum timing and sequencing of radiation in combination with vaccination to achieve a beneficial effect in advanced stage tumors with few side effects is also unclear.

The therapeutic human papillomavirus type 16 (HPV16) vaccines targeting oncoprotein E7 can avoid the immune tolerance against self-antigens and are considered as an ideal choose in the treatments of HPV-associated malignancies^30^. To achieve specific T cell-mediated response, numerous investigations focus on the nanoparticle-based vaccines for co-delivery of antigens and adjuvants^31^. However, limited by proper sizes and other properties, the vaccines rarely get into CD8α^+^ dendritic cells (DCs) located in the deeper paracortex of lymph nodes (LNs) which predominantly mediate the generation of CD8^+^ T cell responses. PEG-PE micelle-based vaccine has a few of the unique properties that is proved to effectively deliver antigens into CD8α^+^ DCs and offer augment antigen-specific CTL responses^32^. We have previously reported that PEG-PE micelle-based vaccines containing specific E7 antigens and monophosphoryl lipid A (MPLA) adjuvant have an impressive antitumor effect, and its combinations with surgery or chemotherapy have been investigated^33^. In the present study, we attempted to address how the combination of RT and PEG-PE micelle-based vaccines could induce an optimal immune response and durable cure in mice bearing solid tumors. In this study, the murine-derived TC-1 cells transformed with HPV16 E6 and E7 were used as a non-metastatic cervical cancer model; 4T1-Luc2 cells transfected by a firefly luciferase cDNA expression vector (Luc2) were used as a metastatic breast cancer model; and 20-mer peptides of HPV-16 E7 (43-62) and 9-mer peptides of Luc2 (160-168) were predicted as producing specific T-cell responses after vaccination and capable of enhancing the immunogenicity in the mammalian host. We found that after radiation, tumors became more immunosuppressive, represented by the increased Tregs population, which was overcome by previous administration of vaccination rather than other treatments. Our findings offer an insight into the optimal timing and sequencing of combination of radiation and vaccination to influence the immune microenvironment of the tumor. Importantly, our proposed combination regimen would be further considered for translation into the clinic, especially for intractable and unresectable tumors.

## Results

### Host immunosuppression of tumor-bearing mice is characterized by progressively reducing the ratio of CD8^+^ T cells to Tregs

Myeloid-derived suppressor cells (MDSCs) and Tregs, both contribute to host immunosuppression and allow peripheral and tumor T-cell inactivation, resulting in tumor immune escape^34^. Our results found that MDSCs (CD11b^+^Gr1^+^) and Tregs (CD4^+^CD25^+^Foxp3^+^) were elevated significantly on days 21 and 28 after tumor implantation (Supplementary Fig. 1a, b), indicating that both Tregs and MDSCs might affect host tumor immune response. When these two populations of cells were detected in TME after RT, the percentage of Tregs but not MDSCs increased significantly (Supplementary Fig. 2f, g). This suggested that Tregs played a predominant role in resistance to radiotherapy. Compared with tumor-free mice, there was a significant reduction in CD8^+^ T cells and the ratio of CD8^+^ T cells to Tregs in the tumor-bearing mice (Supplementary Fig. 1c, d) and this downward tendency was more obvious with tumor progression. Thus, elevating the ratio of CD8^+^ T cells to Tregs to overcome the immunosuppression established by tumors could be a strategy for cancer immunotherapy.

### Radiation increases the number of TILs and enhances immunosuppression in tumor milieu

RT has been reported to increase antitumor immune response. CD45 is known as one leukocyte common antigen^35^, and was used in this study to distinguish leukocytes from non-leukocytes. To understand how RT affects tumor infiltrating lymphocytes (TILs), we measured the MHC-1 molecule expression on non-leukocytes (CD45^−^) and leukocytes (CD45^+^), and found a significantly increased level of MHC-1 expression on both non-leukocytes and leukocytes following radiation, compared with the unirradiated control tumors (Supplementary Fig. 2a–c). In addition, irradiation trigged leukocyte infiltration into tumors, reflected by about 2-fold increase in the proportion of CD45^+^ cells compared with that in the unirradiated tumors (Supplementary Fig. 2d). Although CD8^+^ T cells were significantly elevated in the tumors after radiation (Supplementary Fig. 2e), the percentage of CD8^+^ T cells was low (~0.3%). This raises the question whether radiation induces more Tregs (CD4^+^CD25^+^Foxp3^+^) in tumors. As expected, we found that irradiation led to a 2.1-fold increase in the proportion of Tregs in the tumors compared with the unirradiated tumors (12.13% vs 5.74%, Supplementary Fig. 2f). However, the percentage of MDSCs in tumors was not elevated after radiation (Supplementary Fig. 2g). Our results suggest that the increase in Tregs but not in MDSCs mainly contributed to the radiation-induced immunosuppression in this tumor model.

**Fig. 1:**
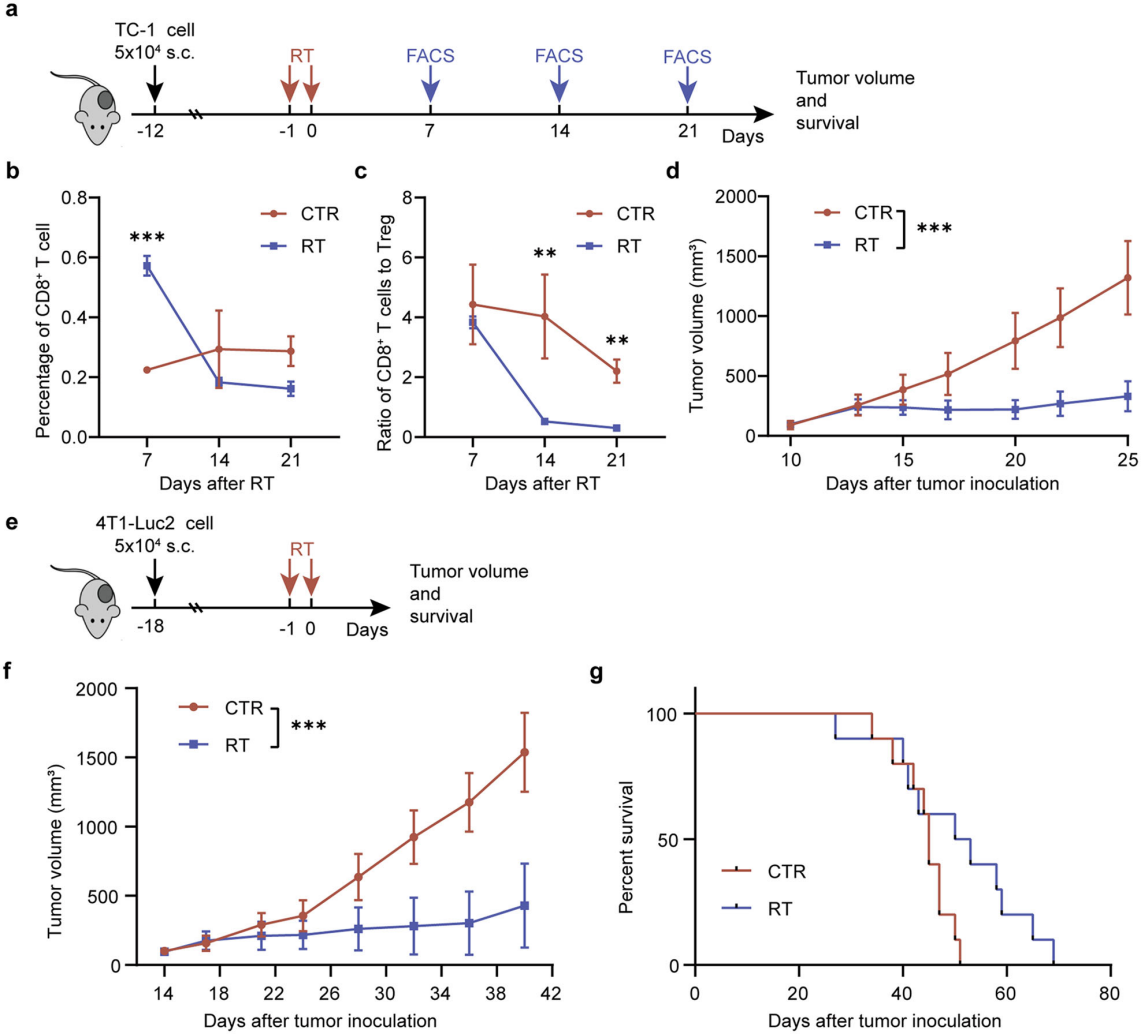
Effect of radiation therapy on local tumor, and survival. **a** Experimental procedure of TC-1 tumor model and treatments. **b**, **c** Flow cytometry analyses (*n* = 4 mice per group). Dynamic changes of CD8 + T cells (**b**) and the ratio of CD8 + T cells to Tregs (**c**) in tumors. **d** Tumor growth curves of mice bearing TC-1 tumors with or without RT treatments (*n* = 10 mice per group). **e** Experimental procedure of 4T1-Luc2 tumor model and treatments. **f** Tumor growth curves of mice bearing 4T1-Luc2 tumors with or without radiation treatment. **g** Survival of mice bearing 4T1-Luc2 tumors treated with or without radiation (*n* = 10 mice per group). Results are expressed as mean ± SD. Experiments were repeated two times and two-tailed *t*-test was used for comparisons of biological replicates. ^*^P < 0.05, ^**^P < 0.01, ^***^P < 0.001.

**Fig. 2:**
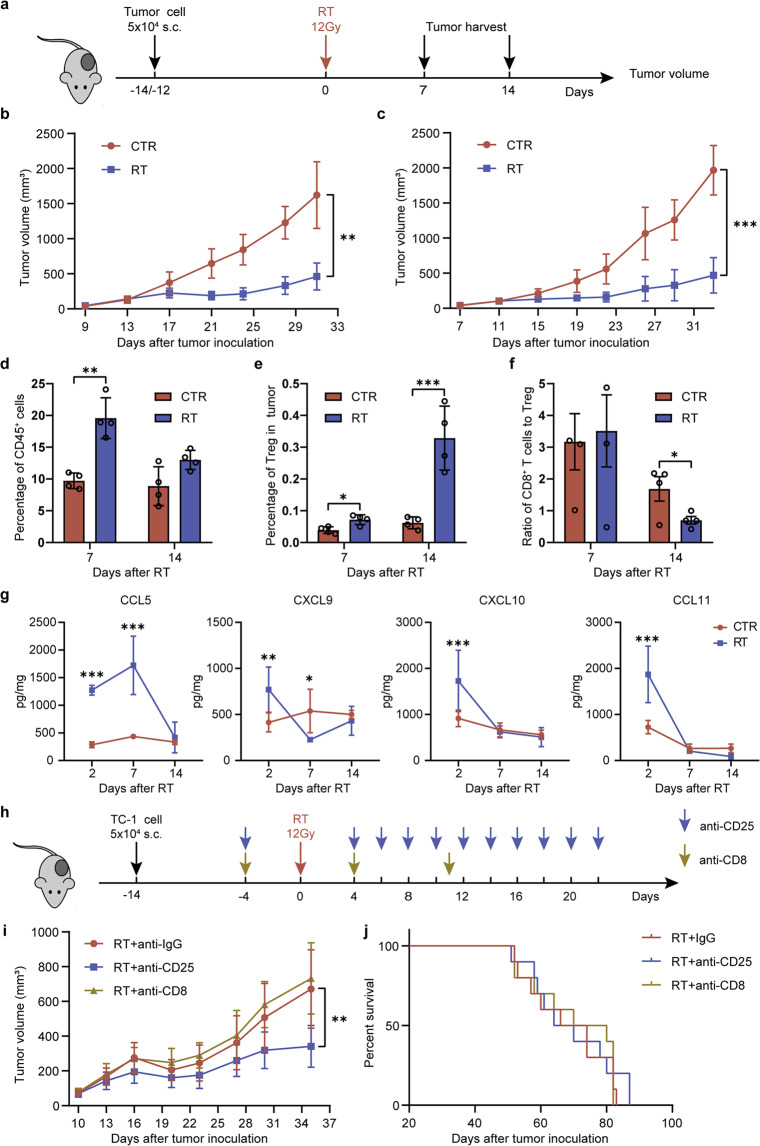
Tregs induced by RT were in charge of tumors relapse. **a** Experimental procedure. **b** Tumor growth curves of TC-1 and **c** 4T1-Luc 2 tumor models. *n* = 10 mice/group. **d**–**f** Flow cytometry analyses in TC-1 tumors. **d** Percentage of dynamic changes of CD45^+^ cells in tumors with time. **e** Percentage of dynamic changes of Tregs (CD45 + CD4 + CD25 + Foxp3 + ) in tumor with time. **f** Ratios of CD8^+^ T cells to Tregs in tumors with time. **g** The level of chemokines in TC-1 tumor (pg/mg protein of tumor tissue). Data are presented as mean ± SD; *n* = 3 to 4 mice/group. **h** Experiment procedure for antibodies depletion. **i** TC-1 tumor growth curves of the mice treated with or without antibodies depletion. **j** Survival curves of the mice bearing TC-1 tumors treated with or without antibodies depletion. *n* = 9 mice/group. Experiments were repeated two times and two-tailed *t*-test was used for two-way comparisons of biological replicates. ^*^P < 0.05, ^**^P < 0.01.

Clinical reports have suggested that tumor relapse following RT increases metastasis and brings poor outcome in cancer patients^36^. Recent evidence has indicated that RT increases suppressive Tregs in the TME^37^. To address whether the Tregs induced by irradiation could be responsible for tumor relapse after radiotherapy, we established two tumor models (TC-1 and 4T1-Luc 2) and irradiation was selectively delivered onto the tumors. Two different radiation treatments were performed using a single dose of 12 Gy or continuous two-fraction doses of 8 Gy (Figs. 1a, e and 2a). The irradiated tumor growth remained with no progression for about 10 days, but afterwards grew rapidly (Figs. 1d, f and 2b, c). Notably, the survival of mice bearing the irradiated tumors was not significantly improved compared with that of mice bearing the unirradiated tumors (Fig. 1g).

Next, we determined the population of TILs in tumors using flow cytometry. Compared with the unirradiated tumors, the percentage of CD45^+^ cells in the irradiated tumors was significantly elevated on day 7 and declined on day 14 after irradiation (Fig. 2d). The number of Tregs on day 14 was three times higher than that on day 7 (Fig. 2e), indicating that Tregs could be responsible for relapse after radiotherapy. Similarly, the irradiated tumors showed a significant reduction in the ratio of CD8^+^ T cells to Tregs with tumor progression (4.52 on day 7 vs 0.70 on day 14; *P* = 0.007) compared with the unirradiated tumors (Fig. 2f). Similar results were obtained in the TC-1 tumor model treated with two-fraction doses of 8 Gy (Fig. 1 b, c). Expression of intratumoral chemokines is reported to be associated closely with the presence of TILs^38^. To determine whether radiation stimulated T-cell response relating to chemokines, we analyzed the level of chemokines on day 2, day 7, and day 14 in tumor tissues after RT. Compared with the untreated control, the level of CCL5 after RT significantly increased on day 2 and day 7 but decreased to the control level on day 14. The levels of CXCL9, CXCL10, and CCL11 showed certain increasing after RT on day 2 and then decreased on day 7 and day 14 (Fig. 2g). These changes in chemokine levels over time suggested that RT induced chemokines to recruit effector T cells in the early stage but failed to maintain it for a durable effect.

Furthermore, the decreased ratio of CD8^+^ T cells to Tregs seems to play a critical role in the suppression of adaptive immune responses in the TME. It showed that depletion of CD8^+^ T cells only slightly abolished the effect of RT, while depletion of CD25^+^ T cells further enhanced the effect (Fig. 2i). However, when compared with RT alone, the depletion of CD25^+^ cells plus RT did not improve survival of mice bearing tumors (Fig. 2j). This suggests that only suppression of the negative regulatory factors such as Tregs is not sufficient to enhance antitumor immunity. Achievement of effective antitumor immunity may require an increase in the ratio of CD8^+^ effector T cells to Tregs.

### Sequential combinations of RT and vaccination alter the ratio of CD8^+^ T cells/Tregs in the tumor milieu

**Fig. 3:**
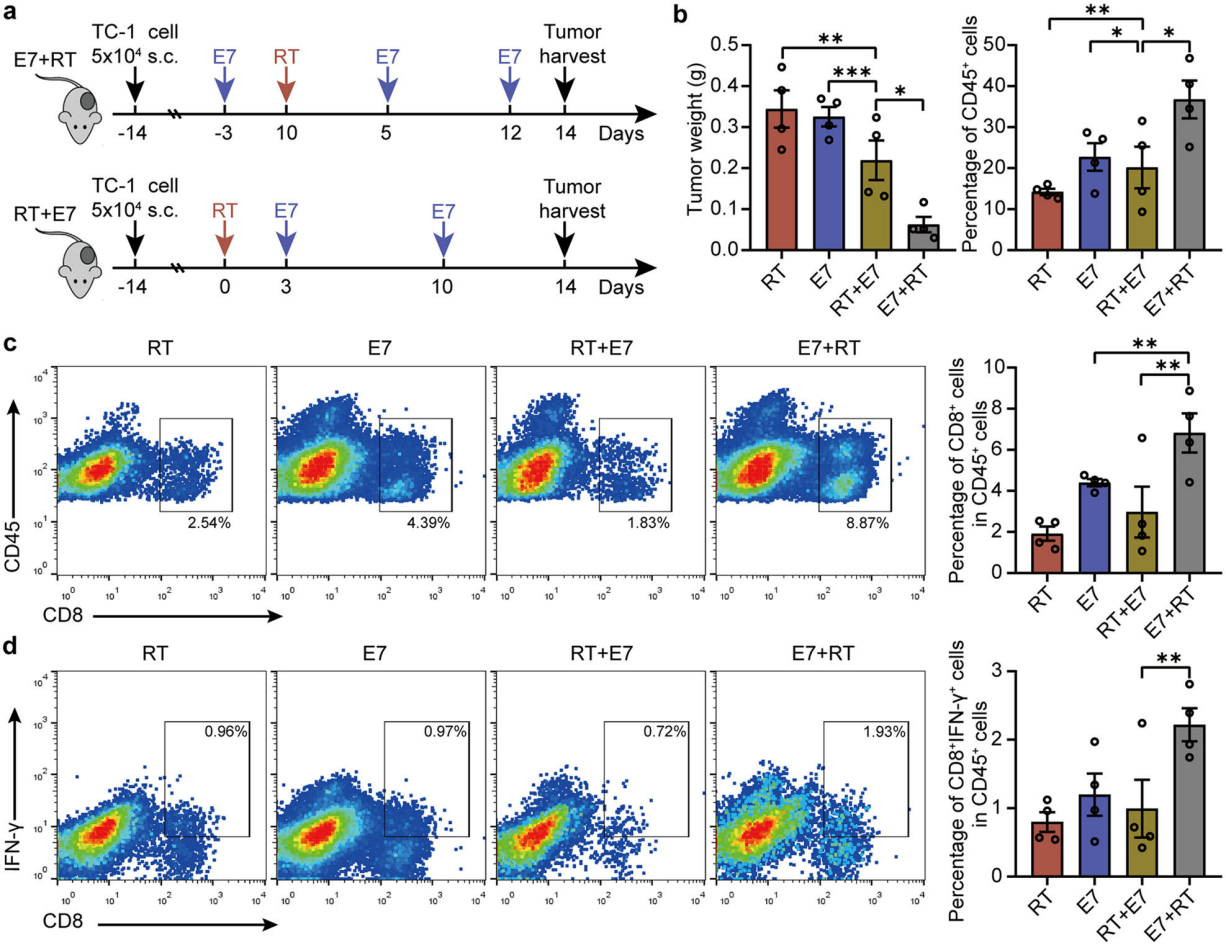
Therapeutic cancer vaccine followed by radiation induced more leukocytes and CD8^+^ effector T cell infiltration in the tumors. Established TC-1 tumors were treated by radiation alone or by the combinations of radiation with vaccines before or after radiation. The tumors were harvested on 14 days after RT. **a** Experimental procedures. **b** The tumor weights (left) and the percentage of CD45^+^ cells (right) in the tumors determined by FACS. **c** The percentage of CD8^+^ cells in CD45^+^ cells in the tumors, and **d** the percentage of CD8^+^IFN-γ^+^ cells in CD45^+^ cells in the tumors analyzed by flow cytometry. Experiments were repeated three times and two-tailed *t*-test was used for comparisons of biological replicates. Data are presented as mean ± SD; *n* = 4 mice/group. ^*^P < 0.05, ^**^P < 0.01, ^***^P < 0.001.

Vaccines can induce multi-epitope, T cell-mediated immunity and stimulate CD8 effector T cells, which offer an effective immune response for cancer immunotherapy^15^. It has been reported that the first dose of vaccine administered prior to irradiation can induce a higher level of CD8^+^IFN-γ^+^ T cells than after irradiation, resulting in a better antitumor activity^29^. While it has also showed that combined irradiation with vaccination only induces modest inhibition of tumor growth, although increasing CD8^+^ T-cell infiltration^20^. In this study, we found that irradiation alone only maintained tumor regression for about 10 days, after which tumor relapsed quickly with a gradually decreased ratio of CD8^+^ T cells/Tregs in the TME. Thus, we assumed that pretreatment with vaccination could induce more effector CD8^+^ T-cell infiltration in tumors, which would overcome the immunosuppressive function of Tregs induced by RT. To address this hypothesis, we compared the isolated tumor weight and population of TILs in TC-1 tumor model using two different sequential combinations of vaccine plus irradiation: vaccination before 3 days of irradiation (E7 + RT) and vaccination after 3 days of irradiation (RT + E7). As the percentage of CD8^+^ T cells and the ratio of CD8^+^ T cells to Tregs in the irradiated tumors dynamically changed with time and relatively stabilized on day 14 after irradiation (Fig. 1b, c), tumors were then harvested and analyzed on day 14 after radiation. The results showed that mice treated with E7 + RT showed a significantly decreased tumor weight in comparison to the mice that received the other three treatments (Fig. 3b). The percentage of CD45^+^ cells in the tumors treated by E7 + RT was elevated 2.47- and 2.85-fold compared with that in tumors treated by RT + E7 (E7 + RT vs RT + E7: 40.5% vs 16.4%) and RT alone (E7 + RT vs RT: 40.5% vs 14.2%), respectively (Fig. 3b). Similar results were found when these experiments were repeated (Supplementary Fig. 3c). Surprisingly, we noted that the CD45^+^ cell populations did not differ between RT alone and RT followed by vaccination (Fig. 3b and Supplementary Fig. 3c). In addition, there was an increased population of CD45^+^MHC-1^+^ cells in tumors treated by E7 + RT compared with RT + E7 and RT alone (Supplementary Fig. 3a) and E7 + RT also significantly upregulated MHC-1^+^ expression in the CD45^−^ cells (Supplementary Fig. 3b). Most interestingly, the percentages of CD45^+^CD8^+^ cells (Fig. 3c) and effector CD8^+^IFN-γ^+^ T cells (Fig. 3d) in the tumors treated by E7 + RT was about 4-fold higher than in the tumor treated by RT + E7 or by RT alone. However, when compared with RT alone, RT + E7 treatment did not alter the percentage of CD8^+^ cells and CD8^+^IFN-γ^+^ T cells of intratumoral CD45^+^ cells (Fig. 3c, d and Supplementary Fig. 3d, e).

To further understand whether the order of combination of RT and vaccination influences the qualities of antitumor effect, we compared the immune effects of two combination therapies with the same times of vaccination and irradiation in a different order. The results showed that, after mice received two doses of vaccine and one irradiation, E7 + RT treatment induced much stronger immune response in tumor than that of RT + E7 treatment, characterized by significant improvements in the percentages of CD45^+^ cells, CD8^+^ T cells in CD45^+^ cells, CD8^+^IFN-γ^+^ cells, and the ratio of CD8^+^ T cells to Tregs (Supplementary Fig. 4). These results suggested that the order of combination between RT and vaccination had distinctive immune-mediated antitumor effects.

To determine whether E7 vaccine induced an increase in tumor antigen-specific CD8^+^IFN-γ^+^ CTLs, we measured the percentage of E7-specific CTLs in lymphocytes in normal mice after two doses of E7 vaccine. HPV16 E7_49-57_ peptides were used to stimulate lymphocytes for 6 h. Compared with the non-vaccinated control mice, there showed significantly higher levels of CD8^+^ T cells and E7-specific CTLs (Supplementary Fig. 5).

**Fig. 4:**
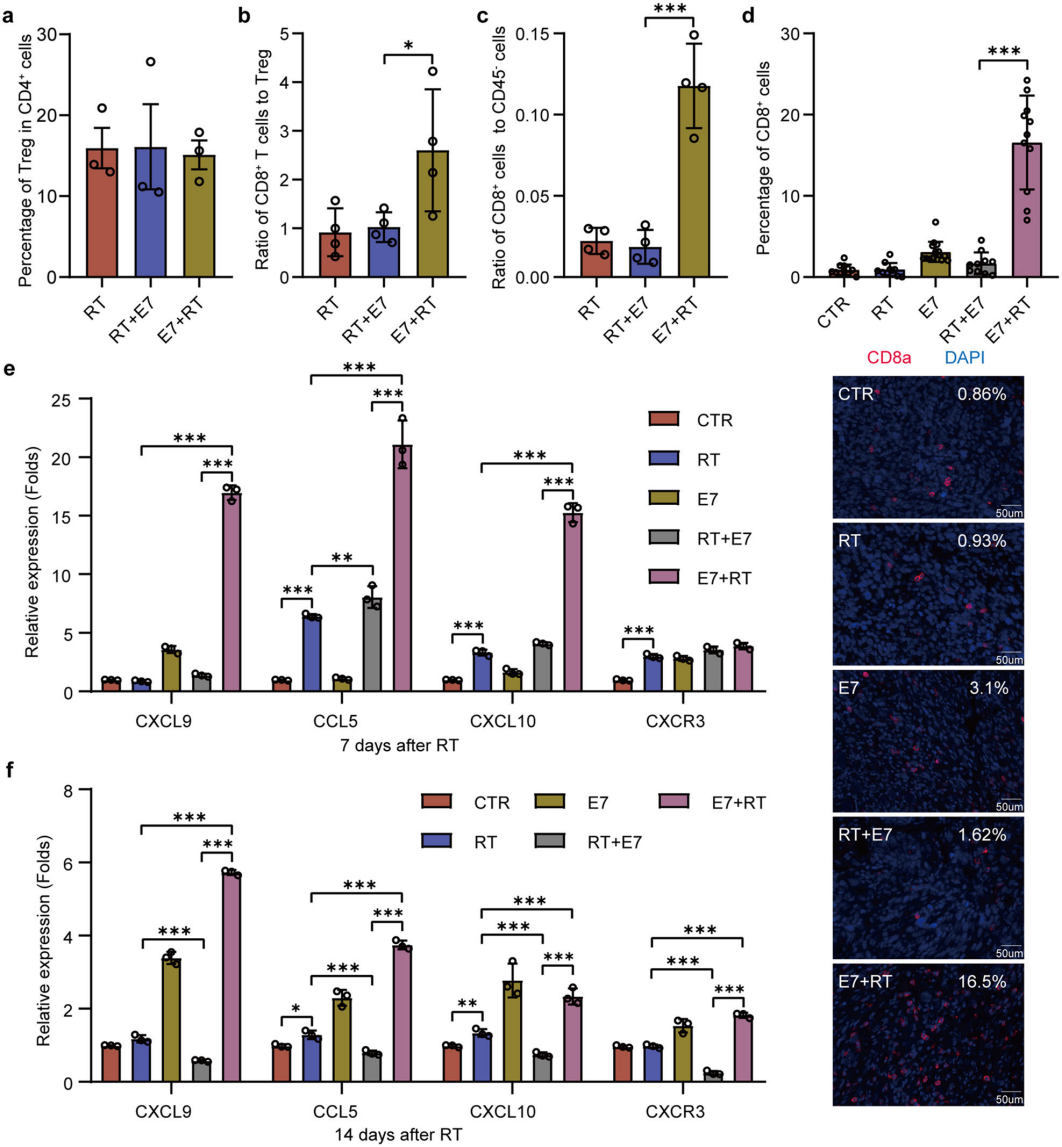
Tumors treated by the combinations of E7 vaccines before or after radiation showed bigger difference in the ratios of CD8^+^/Treg cells. Local established tumor treatments were followed by the experimental procedures described as in Fig. 3a. On days 7 and 14 after radiation, tumors were collected for flow cytometry, immunofluorescent staining, and RT-PCR analyses. **a** Percentage of Tregs in CD4^+^ cells analyzed using flow cytometry. **b** The ratio of CD8^+^/Tregs in tumors analyzed using flow cytometry. **c** The ratio of CD8^+^/CD45^−^ cells in tumors analyzed using flow cytometry. **d** From each group 3 tumors were used for the immunofluorescent staining of CD8. Slides were incubated with anti-CD8 primary antibodies overnight in 4 °C and then with corresponding secondary antibodies (RED), then stained the nucleus with DAPI (BLUE). 10 to 12 fields of view in each treatment group were imaged and analyzed (Upper). The pictures were merged by image software (Lower). Percentage of CD8^+^ positive staining cells was analyzed using Image-Pro Plus software. **e**, **f** Whole tumor RNAs were isolated from the tumors of treated mice. Real-time PCR measurement of the expression of CXCL9, CCL5, CXCL10, CXCR3 in tumors after radiation for 7 days (**e**) and 14 days (**f**). Data were presented as mean ± SD; *n* = 3 to 4 mice/group. Experiments were repeated three times and two-tailed *t*-test was used for comparisons of biological replicates. ^*^P < 0.05, ^**^P < 0.01, ^***^P < 0.001, ^****^P < 0.0001.

Tregs in tumors are considered to be primary inducers of immunosuppression in the TME, abundant and widespread, and associated with poor prognosis^39^. In this study, we demonstrated that radiation induced more accumulation of Tregs in the TME compared with the untreated control (Fig. 2e). One concern was whether combination therapy, compared with radiation alone, increased the number of Tregs in the TME. By analysis of CD4^+^CD25^+^Foxp3^+^ cells in the tumor tissues, we found that the combined therapies (E7 + RT and RT + E7) did not change the intratumoral percentage of Tregs (Fig. 4a), indicating that E7 + RT therapy could induce a higher ratio of CD8^+^ T cells/Tregs in the tumors that contributes to its better antitumor effect. As expected, the ratio of CD8^+^ T cells/Tregs in the tumors treated with E7 + RT was significantly higher than that in the tumors treated with RT + E7 and RT alone (Fig. 4b). Furthermore, compared with that in RT and RT + E7 groups, the higher percentages of CD8^+^ T cells, CD8^+^IFN-γ^+^ cells (Supplementary Fig. 6a–d), and a higher ratio of CD8^+^ T cells to Tregs in lymph node and spleen lymphocytes (Supplementary Fig. 6e, f) were found in E7 + RT group. In addition, among the treatments, E7 + RT showed the highest ratio of CD8^+^ T cells to CD45^−^ cells in tumors (Fig. 4c and Supplementary Fig. 3f). The immunohistochemistry imaging and statistical data also provided visible evidences that the percentage of CD8^+^ positive staining cells in the tumor sections treated by E7 + RT (16.5%) was much higher than the other three treatments (Fig. 4d and Supplementary Fig. 7). Our results support that increasing the intratumoral ratio of CD8^+^ T cells/Tregs could be a marker of improved immune response and tumor shrinkage.

As previous studies have reported that recruitment of immune cells into tumors is mediated by chemokine levels, we then investigated whether chemokines were induced by sequential combination of RT and vaccination in the TC-1 tumor model. It showed that the mRNA levels of chemokines, including CCL5, CXCL9, CXCL10, and their receptor CXCR3, were higher in tumors treated with E7 + RT compared with other treatments on day 7 (Fig. 4e) and day 14 (Fig. 4f). Taken together, our results demonstrated that combination of vaccination followed by RT significantly induced the systemically high production of IFN-γ in CD8^+^ T cells; the enhanced MHC-1 expression on the surface of tumor cells and lymphocytes; and the increased chemokine production in the TME that attracted more effector CD8^+^ T-cell infiltration into the tumor milieu, thus leading to a favorable immune microenvironment.

**Fig. 5:**
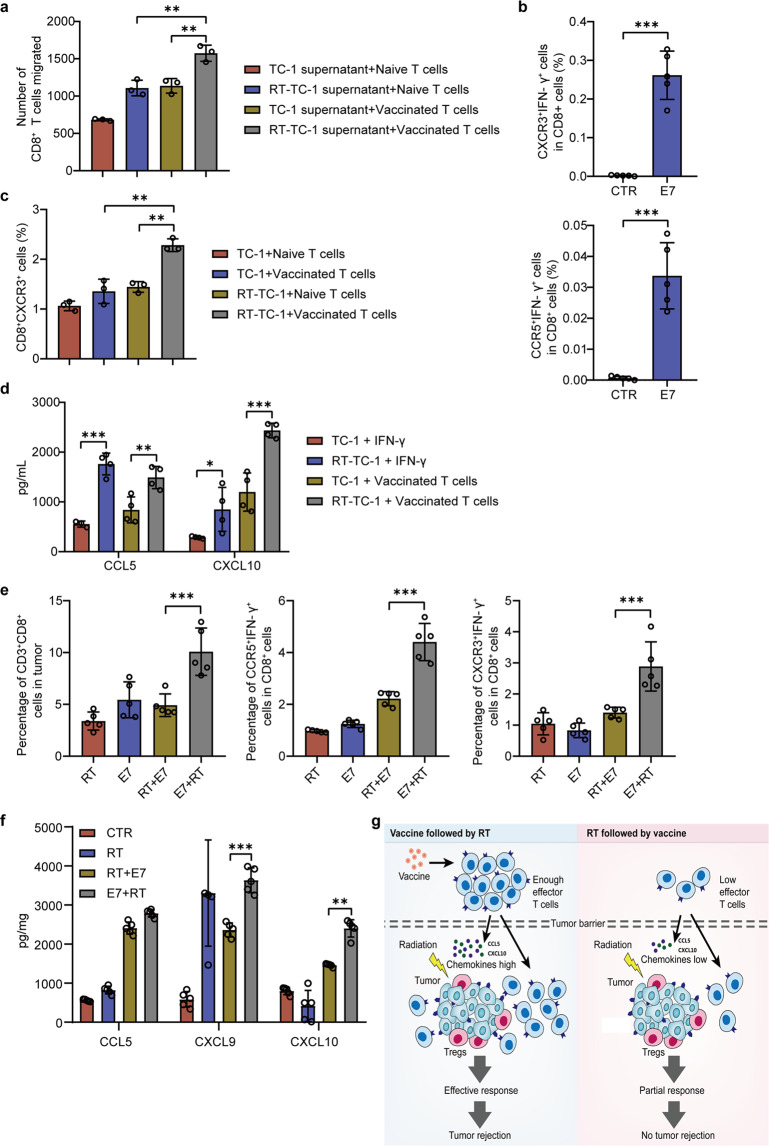
Vaccine- and radiation-induced chemokines promoted the migration of the vaccination activated CD8+ T cells in vitro. For study of CD8^+^ T cells migration, irradiation (8 Gy, X-ray) was applied to TC-1 cells (5 × 10^4^ cells/mL), and mice received one dose of E7 vaccine (10 μg/mouse) followed by making single T cell suspension for lymph nodes after 7 days of vaccination. **a**–**f** Flow cytometry analyses. **a** The number of T cells migrated in lower chamber of transwell plates were counted by flow cytometry. Supernatants (2 × 10^4^ cells/mL, 1 mL) collected from TC-1 cells treated with or without irradiation were filled in the lower chamber and T cells (1 × 10^6^ cells/mL, 200 μL) were added in upper chamber, and incubated for 24 h at 37 °C. **b** After 7 days vaccination, T cells were isolated from the inguinal lymph nodes. The cells were seeded and stimulated with E7_49-57_ peptide (5 μg/mL) and added BFA (1 μg/mL) for 6 h. The percentage of CXCR3^+^IFN-γ^+^ cells (upper) and CCR5^+^IFN-γ^+^ cells (lower) in CD8^+^ T cells was analyzed by flow cytometry. **c** TC-1 cells (2 × 10^4^ cells/mL, 1 mL) treated with or without irradiation were co-cultured with T cells (1 × 10^6^ cells/mL, 200 μL) from the mice vaccinated or not, for 24 h incubation, the percentage of CD8^+^CXCR3^+^ T cells was measured by flow cytometry. **d** TC-1 cells (2 × 10^4^ cells/mL, 1 mL) treated with or without irradiation were co-cultured with T cells (1 × 10^6^ cells/mL, 200 μL) from the vaccinated mice or added with 100 ng/mL of IFN-γ^+^ for 48 h incubation, the supernatants were collected for CCL5 and CXCL10 chemokines assay. **e** Mice bearing TC-1 tumors (tumor volume about 150 mm^3^) were treated with RT, vaccination (**E7**), RT followed by vaccination (RT + E7), and vaccination followed by RT (E7 + RT) for 14 days. Mice were sacrificed and tumors were dissected for further detection. The percentage of CD3^+^CD8^+^ T cells in tumor (left), CCR5^+^IFN-γ^+^ cells (middle), and CXCR3^+^IFN-γ^+^ cells (right) in CD8^+^ T cells was analyzed by flow cytometry. **f** The regulatory mechanism of the recruitment of CD8^+^ T cells by chemokines CCL5 and CXCL10 after different combinations of vaccination and RT. **g** A schematic diagram of different regulatory mechanisms of the recruitment of CD8^+^ T cells by chemokines CCL5 and CXCL9/10 after the combinations of vaccination and RT in reverse order. Data were presented as mean ± SD; *n* = 5 mice/group. Experiments were repeated three times and two-tailed *t*-test was used for comparisons of biological replicates. ^*^P < 0.05, ^**^P < 0.01, ^***^P < 0.001, ^****^P < 0.0001.

We attempted to establish the reason why combination of E7 + RT rather than RT + E7 caused more effector T-cell infiltration into the tumors. Previous studies have shown that IFN-γ produced by CD8^+^ T cells can stimulate the expression of CXCR3 ligands CXCL9 and CXCL10 in epithelial cells, which in turn leads to CD8^+^ T-cell proliferation and migration^40,41^. We examined CD8^+^ T-cell migration governed by chemokines in vitro; the cells were isolated from lymph nodes of normal mice with or without vaccination. The transwell assay showed an enhanced migration of CD8^+^ T cells when the cells were co-cultured with the supernatant of irradiated TC-1 tumor cells or when CD8^+^ T cells were from vaccinated mice (Fig. 5a). Flow cytometry showed that expression of CXCR3 and CCR5 on IFN-γ-producing CD8^+^ T cells was significantly enhanced in lymphocytes isolated from pre-vaccinated mice (Fig. 5b). When T cells derived from vaccinated mice were co-cultured with irradiated TC-1 tumor cells, the population of CXCR3^+^CD8^+^ T cells was higher than that when co-cultured with non-irradiated TC-1 cells, or when non-vaccinated T cells were co-cultured with irradiated TC-1 cells (Fig. 5c). To investigate whether vaccination-induced IFN-γ could stimulate tumor cells to produce some chemokines, and these chemokines in turn activated CXCR3 signaling in T cells, E7-vaccinated lymphocytes were co-cultured with TC-1 tumor cells pretreated with or without irradiation. Compared with TC-1 without irradiation, TC-1 tumor cells pretreated with irradiation were induced greater production of CCL5 and CXCL10 by IFN-γ. However, when compared with the irradiated TC-1 cells treated by IFN-γ, the addition of the vaccinated T cells to the irradiated TC-1 cells significantly increased CXCL10 production but not CCL5 (Fig. 5d). To further investigate whether the recruitment of CD8^+^ T cells was induced in E7 + RT treatment in vivo, the population and phenotypes of lymphocytes and expression levels of chemokines were analyzed in the irradiated tumors. A three-step FACS gating strategy was used for the sorting of CD8^+^CXCR3^+^IFN-γ^+^ and CD8^+^CCR5^+^IFN-γ^+^ cells from tumor tissue suspension (Supplementary Fig. 8). As shown in Fig. 5e, f, compared with other treatments, E7 + RT treatment induced the increases of CD3^+^CD8^+^ T cells recruitment, percentages of CXCR3^+^IFN-γ^+^ cells and CCR5^+^IFN-γ^+^ cells in CD8 + T cells, and expression levels of chemokines, such as CCL5 and CXCL9/10. Our results demonstrated that the elevated levels of CCL5 and CXCL10 produced by the irradiated tumors might attract the vaccinated CD8^+^ T cells with a high expression of IFN-γ^+^CXCR3^+^ into the tumor, resulting in an increased ratio of CD8^+^ T cells/Tregs in the tumor milieu. The different regulatory mechanism of the recruitment of CD8^+^ T cells by chemokines CCL5 and CXCL10 after different order of vaccination and RT is shown in Fig. 5g. In addition to chemokines, the enhanced MHC class I expression in leukocytes and non-leukocytes could also play an important role in the mediation of immune response in the irradiated tumor milieu.

### Vaccination prior to RT is effective for the eradication of solid tumors

**Fig. 6:**
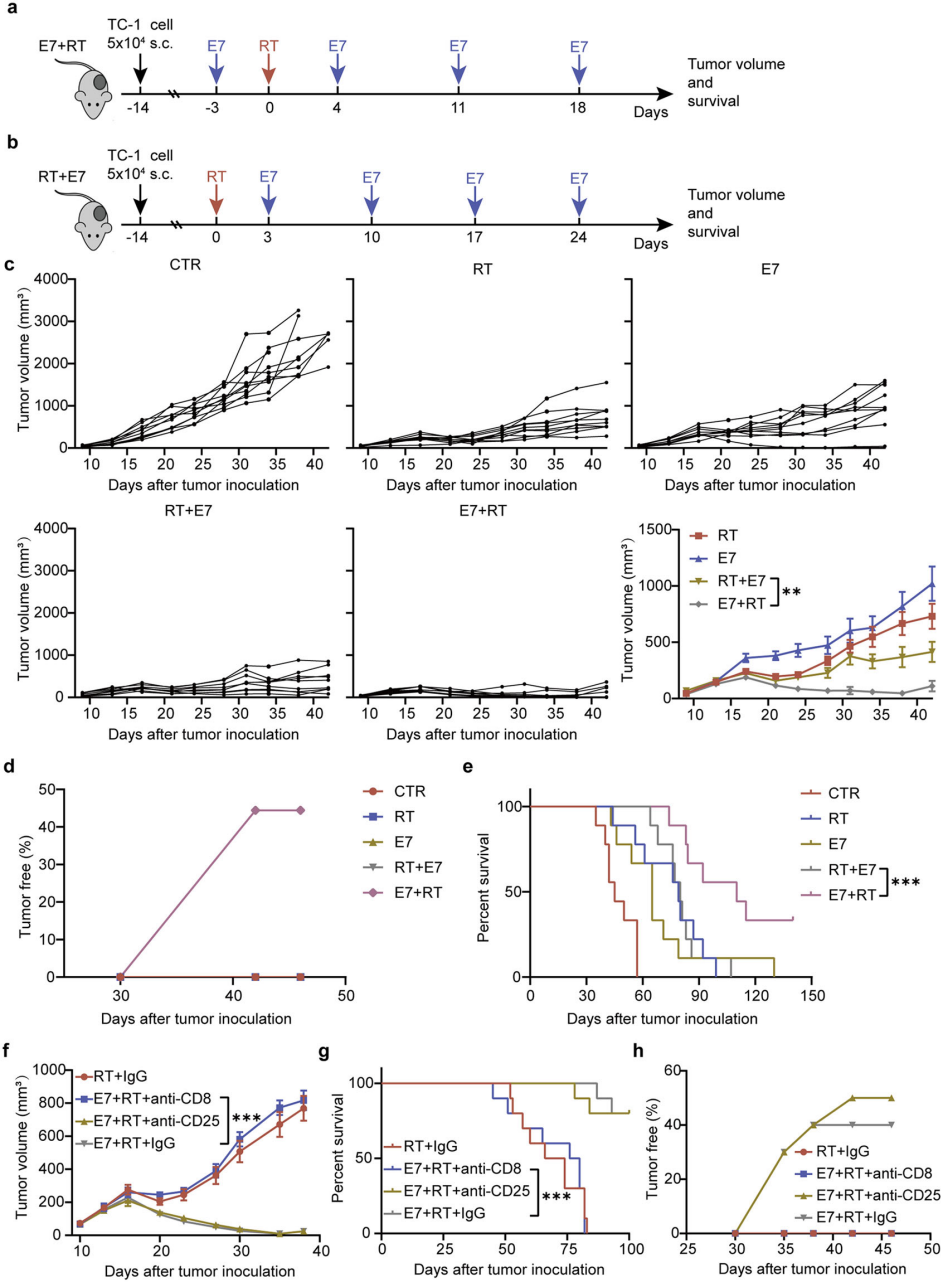
Sequences of vaccine administration and tumor irradiation were crucial to produce durable antitumor effects. **a**, **b** Experimental procedures. **a** Vaccination followed by RT (E7 + RT): tumor-bearing mice received the first dose of E7 vaccine (5 μg per mouse) after tumor cells injection for 11 days, then the tumors were irradiated on day 14, another three doses of E7 vaccines were administrated subcutaneously (sc.) on days 4, 11, and 18 after RT. **b** RT followed by vaccination (**RT** + **E7**): tumors were irradiated with single dose of radiation on day 14 after tumor cells injection and then followed by four doses of E7 vaccines sc. on days 3, 10, 17, and 24 after RT. **c** Individual tumor growth curves and average tumor volumes with time, untreated tumor-bearing mice as control (CTR), 9 mice for each treatment. Tumors isolated from mice after treatment on day 14 were photographed as 4–5 mice for each treatment. **d** Percentage of the tumor-free mice that received with different treatments, only E7 + RT treatment showed 4 mice with tumor rejection. **e** Survival curves of the TC-1 tumor-bearing mice with various treatments. **f** Depleting experiments with antibodies. Tumor-bearing mice treated by E7 + RT with anti-CD25 (10 doses, 200 μg/mouse) or anti CD8 (3 doses, 200 μg/mouse) antibodies, IgG as control, for details, see the Methods and the schematic shown in Fig. 2h. **g** The percentages of mice survival and **h** the tumor-free mice followed various treatments, 10 mice for each treatment. Results are presented as mean ± SD. Experiments were repeated two times and two-tailed *t*-test was used for two-way comparisons of biological replicates. ^*^P < 0.05, ^**^P < 0.01, ^***^P < 0.001, ^****^P < 0.0001.

It is rare for immunotherapy to eradicate solid tumors in syngeneic immunocompetent mice^42^. We also have found that solid tumors with volumes above 100 mm^3^ cannot be cured by vaccination alone^33^. E7 + RT rather than RT + E7 induced a high ratio of CD8^+^ T cells/Tregs and massive infiltration of tumor-associated, antigen-specific CD8^+^ T cells into the tumor milieu (Figs. 3 and 4). Thus, we speculated that E7 + RT would produce durable antitumor effects on solid tumors that are larger than 100 mm^3^ (about 150 mm^3^). To investigate the efficacy of different sequential combination therapies on mice bearing localized tumors, two strategies were used (Fig. 6a, b). Compared with the untreated tumors, those treated with RT or E7 vaccine alone showed delayed growth for a short period of ~10 days and then started to grow (Fig. 6c). Although RT + E7 treatment significantly delayed tumor growth, compared with RT alone, robust tumor regression and durable cure were not observed (Fig. 6c). In contrast to RT + E7 treatment, E7 + RT induced robust tumor regression and durable cure in 44.4% of mice bearing TC-1 tumors, and all the long-term survivors rejected tumors after re-challenge (Fig. 6c, d). E7 + RT treatment also brought TC-1 tumor-bearing mice a longer survival than the other three treatments (Fig. 6e). To confirm whether tumor rejection induced by E7 + RT was dependent on CD8^+^ T cells or Tregs in the TME, we selectively depleted Tregs or CD8^+^ T cells using anti-CD25 and anti-CD8 monoclonal antibodies, respectively. Depletion of CD8^+^ T cells reduced the efficacy of E7 + RT to that of RT alone. Depletion of CD25^+^ T cells had no influence on the efficacy of E7 + RT (Fig. 6f, g) and improved tumor regression from 40% to 50% (Fig. 6h).

### Vaccination prior to RT has an anti-metastatic effect on triple negative breast cancer

**Fig. 7:**
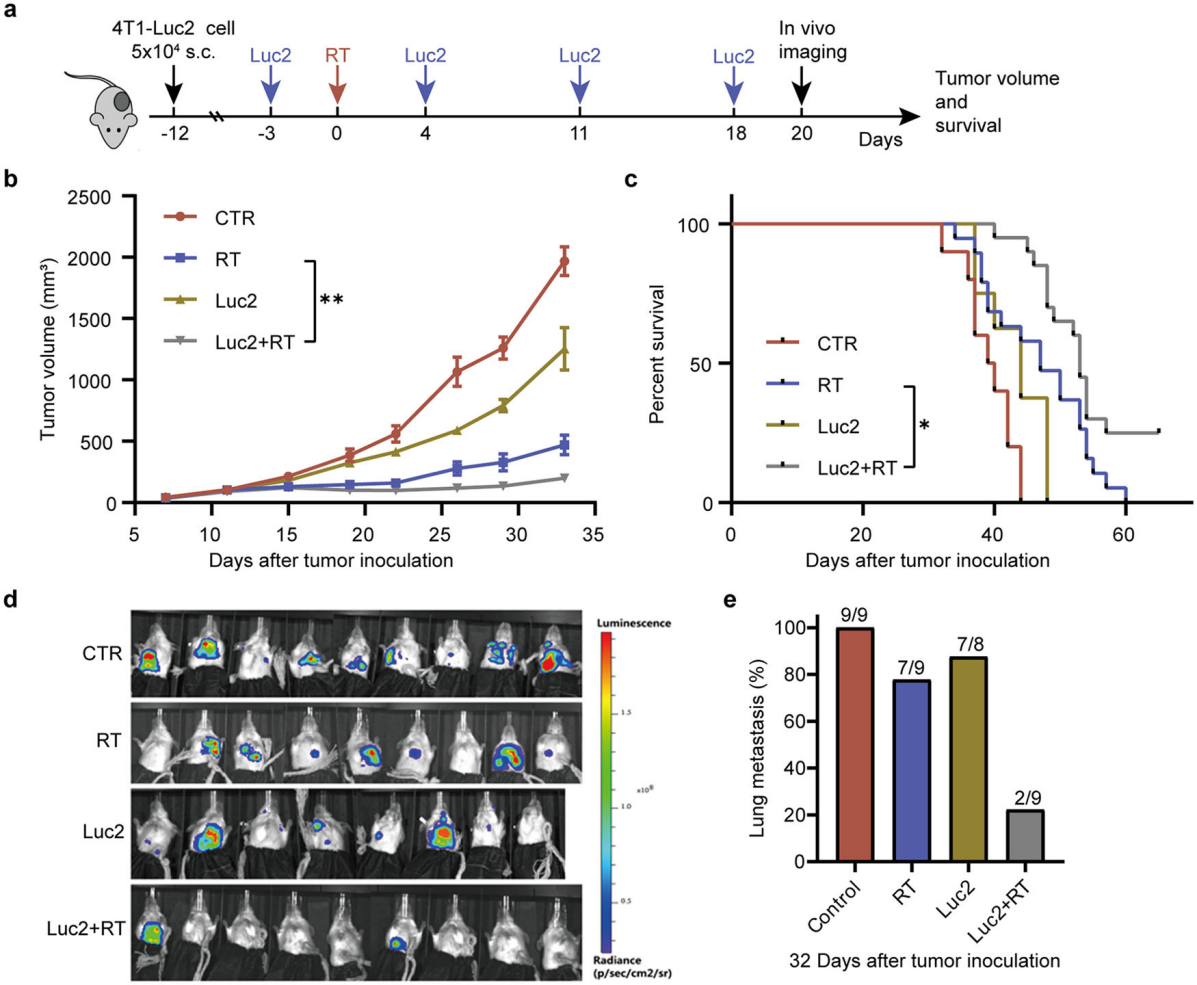
The combination therapy of Luc2 vaccine followed by RT effectively prevented lung metastases of 4T1-Luc 2 tumor. 4T1-Luc 2 cells (5 × 10^4^ cells/mouse) were inoculated on the forth fat-pad of BALB/c mice. **a** Experimental procedure: tumor-bearing mice received the first dose of Luc2 vaccine (6 μg per mouse) on day 9 after tumor cells injection, then on day 12 the tumors were irradiated, another three doses of E7 vaccines were administrated subcutaneously (sc.) on day 4, 11, and 18 after RT. **b** Tumor volume in 4T1-Luc2 tumor-bearing mice over time. **c** Survival curves of the 4T1-Luc2 tumor-bearing mice with various treatments. Results are presented as mean ± SD, 8–9 mice for each treatment. **d** Bioluminescence imaging of 4T1-Luc2 breast cancer lung metastasis on day 23 after RT in vivo using IVIS. **e** The incidence of lung metastases of the mice received various treatments. Experiments were repeated two times and two-tailed *t*-test was used for two-way comparisons of biological replicates. ^*^P < 0.05, ^**^P < 0.01, ^***^P < 0.001, ^****^P < 0.0001.

Triple negative breast cancer cells have an ability to escape immune surveillance and spread to other organs from their primary locus. To investigate whether the potent immunity induced by vaccination followed by RT effectively prevented breast cancer metastasis, we used the 4T1-Luc2 tumor model with a high risk of invasion and metastasis to mimic human triple negative breast cancer. We selected 4 Luc2 epitopes (Luc2_32_, Luc2_160_, Luc2_293_, and Luc2_446_) and analyzed their ability to stimulate antigen-specific CTLs. As Luc2_160_ induced the highest percentage of CTLs (Supplementary Fig. 9), this peptide was used as the antigen to prepare the micelle-based Luc2 vaccine in subsequent combination therapies. We measured primary tumor volume, lung metastasis, and survival in mice bearing 4T1-Luc2 tumors treated with Luc2 vaccine followed by RT (Fig. 7a). Compared with no treatment, Luc2 vaccine or RT alone delayed primary tumor growth within a short period of time, while combination of Luc2 followed by RT significantly suppressed tumor growth over a long time (Fig. 7b). Furthermore, in contrast to monotherapy with RT or Luc2 vaccine, combination therapy greatly impeded lung metastases (Fig. 7d, e). Survival of mice was significantly prolonged in the combination therapy group compared with the other treatment groups (Fig. 7c). These results demonstrated that combination of vaccination followed by radiation could induce a robust endogenous immune response to overcome advanced tumors with high metastatic potential.

## Discussion

An increasing number of clinical tumor specimens have raised the question of why cancers grow progressively despite increased inflammatory and immune responses in patients. In this study, we showed that, as tumor size increased in mice, the number of CD8^+^ T cells decreased and the number of Tregs increased in the tumor and spleen. This indicated that immunosuppression was reinforced with tumor progression. Our observations in mouse models are in agreement with clinical studies which shown that TILs in patients are functionally defective^43^, indicating tumor-induced immunosuppression.

RT targeted against solid tumors is considered to induce the release of antigens and the enhanced infiltration of activated T cells^10^. However, there are increasing evidences that RT changes immunity from enhancing to suppressing in the TME, in which Tregs have been long suspected to be a major player for inhibition of antitumor immunity^44–46^. An analysis of 124 published articles showed that a high percentage of CD8^+^CD45RO^+^ T cell and a low percentage of Tregs in melanoma and breast carcinoma were positively associated with the prognosis of the patients^8^. Tregs are reported to be more resistant to radiation than other lymphocytes are, resulting in their preferential increase^47^. Checkpoint inhibitors, such as anti-PD-L1 and anti-CTLA4 antibodies, are widely used as adjuncts to radiation for restoring T-cell activation and enabling T cells to control cancer progression^16,17^. Although these combined treatments improve antitumor immune responses in the irradiated tumors, resistance usually arises because of T-cell exhaustion. Unlike that in sensitive tumors, CD8^+^ T /Tregs ratio is decreased in resistant tumors. Here, we demonstrated that the CD8^+^ T /Tregs ratio decreased with tumor progression and the ratio became lower after radiation (Supplementary Figs. 1 and 2), suggesting that CD8^+^ T /Tregs ratio plays important role in regulating tumor growth following RT.

MDSCs have been reported to facilitate tumor progression by suppressing immune responses^8^. The population of MDSCs can be induced^48^ or reduced^49^ in tumors after RT, suggesting that MDSCs may play a paradoxical role in regulating irradiated tumors. In our TC-1 tumor model, the MDSC population did not change as much as Tregs after radiation. The depletion of CD25^+^ cells induced a stronger effect on tumor growth inhibition in the irradiated tumors, indicating that Tregs account for a negative effect on radiation. However, anti-CD25 for depleting Tregs, combined with RT, did not induce durable cure, indicating that effective antitumor immunity mainly depended on CD8^+^ effector T cells.

Although cancer therapeutic vaccines have shown promise in preclinical studies, their clinical effects have been limited in cancer patients^50^. One possible reason for the inability of vaccines to eliminate cancer cells is that antitumor T cells hardly enter tumor tissues, particularly in solid tumors. Hence, for treatment of established tumors, combinations of vaccines with other therapies such as RT are indispensable. In this study, we demonstrated that vaccination followed by RT induced effective antitumor immunity both in malignant TC-1 and 4T1-Luc2 solid tumors. RT alone, however, did not achieve beneficial antitumor immunity because of the large number of Tregs induced, which may lead to mutually reinforcing immunosuppression. As vaccination provided ample CD8^+^ T cells, the combination of vaccine with RT was expected to induce a high ratio of CD8^+^ T cells/Tregs in the TME. The increased infiltration of effector CD8 cells and reduction of suppressive Treg cells in the TME are significant for cancer immunotherapy.

Human cancer is so complex that it is difficult to explore effective vaccines. As shown in this study, E7 and Luc2 antigens overexpressed in high level of tumor cells were proved efficient in tumor elimination and survival extension. It gives us a hint that if an antigen specifically overexpressed in certain cancer, such as HER2 in breast cancer and MUC1 in lung cancer, the epitopes could be presented and effectively induce human T cells immunogenicity. Therefore, screening and exploring specific antigens of human tumor cells might be important in vaccine clinical translation. Clinical observations have demonstrated that patients with certain cancers obtain more benefit from immunotherapy if they have received RT^51^. Previous studies have demonstrated that the timing of radiation or chemotherapy combined with immunotherapy is critical for the elimination of established tumors^52^. The antitumor effects of immunotherapy usually be feeble due to the action of immunosuppressive cells in TME in many solid cancers. The utilization of vaccine plus radiation for synergistic antitumor therapy is gaining much attention^27,28^. Thus, better understanding of how the immune molecules respond after treatment will help to optimize the timing and sequencing of RT and vaccination to achieve an ideal therapeutic effect. Here, we uncovered that, compared with RT followed by vaccination, vaccination followed by RT induced a higher level of chemokines and CD8^+^/Tregs ratio in tumors. Our study was consistent with the reports that radiation promotes immune cell recruitment through increasing chemokine levels^53^. This indicated that vaccination followed by RT increased production of chemokines in favor of recruitment of CD8^+^ T cells into the irradiated tumors. Vaccination enhanced the expression of CCR5^+^ IFN-γ^+^ and CXCR3^+^IFN-γ^+^ on CD8^+^ T cells, which led to a significant increase in CCL5 and CXCL9/10 secretion from irradiated tumor cells. In turn, the irradiated tumor cells significantly enhanced migration of CCR5^+^ CD8^+^ and CXCR3^+^CD8^+^ T cells to the TME (Fig. 5).

When CD8^+^ T cells were depleted, the synergistic antitumor effects of vaccination followed by RT were totally abolished (Fig. 6), indicating the dominant function of CD8^+^ effector T cells in the combination therapy. When vaccines were administrated prior to RT, the abundant CD8^+^ effector T cells offset the increased Tregs induced by radiation, resulting in a high ratio of CD8^+^/Tregs in tumors, and markedly enhanced antitumor effects. In contrast, when RT was administered before vaccination, the abundant irradiation-induced Tregs countered the function of the vaccination-induced CD8^+^ effector T cells, leading to a low ratio of CD8^+^/Tregs in the tumors. Our study elucidates the reason for the advantages of sequential vaccination combined RT for treatment of tumors, through inducing robust immune responses.

A detailed understanding of the relationships between tumor and immune cells in the TME after combined treatment is essential to optimize the therapeutic regimen for cancer patients. In this study, we demonstrated that CD8^+^ effector T cells prepared in advance is an important component for counteracting radiation-induced Tregs and essential for robust tumor regression and durable cure. We demonstrate how to manipulate TILs, especially the ratio of CD8^+^ T cells/Tregs in the TME, by simply altering the sequence of RT and vaccination. These therapeutic regimens are suitable for clinical translation and presumably cure many tumors viewed as intractable and unresectable.

## Methods

### Experimental animal and cell lines

Female, 6-8 weeks old C57BL/6 J and BALB/c mice were obtained from Charles River Laboratory Animal Technology Co. (Beijing, China). TC-1 and 4T1-Luc 2 cells were cultured using complete RPMI 1640 (Gibco) medium with 10% Fetal Bovine Serum (HyClone), 100 U/mL penicillin (Beyotime), and 100 U/mL streptomycin (Beyotime) and incubated at 37 °C, with 5% CO_2_.

### Preparation of PEG-PE micelle-based vaccine

The PEG-PE micelle-based vaccine was prepared by film-rehydration method. Briefly, PEG-PE was dissolved in chloroform. MPLA was dissolved in chloroform and methanol with a volume ratio of 2:1. HPV16 E7_43-62_ peptides and Luciferase Luc2 peptides were dissolved in methanol. Then, the components of mixture were PEG-PE, MPLA and peptides in a mass ratio of 250: 2.5:10. The organic solvents were removed using a rotary evaporator to form antigen peptide-containing lipid film. Then the lipid film was hydrated with sterile deionized H_2_O at 50 °C for 30 min under the protection of nitrogen.

### Design and screening for antigens chosen

HPV16 E7 (43-62, GQAEPDRAHYNIVTFCCKCD) were used as HPV16 E7 antigens^33^. The Luc2 antigen epitopes (32-40, 160-168, 293-301, 446-454) were selected by using the website http://www.syfpeithi.de/bin/MHCServer.dll/EpitopePrediction.htm. To choose the effective Luc2 antigen epitopes, lymphocytes from inguinal draining lymph nodes of C57BL/6 J mice vaccinated with Luc2 vaccines for 1 week were isolated. The cell suspensions were re-stimulated in U-bottom 96-well plates with 5 μg/mL corresponding peptides for 6 h in the presence of Brefeldin A (5 μg/mL). After blocking with an anti-FcγR mAb (2.4G2), the cells were stained with antibodies against CD8α (clone 53–6.7) before fixation/permeabilization and intracellular staining for IFN-γ (clone XMG1.2). The samples were then detected by BD FACS Caliber flow cytometer (BD Biosciences, San Jose, CA, USA) and the data were analyzed using the FlowJo software (Tree Star Inc., Ashland, OR, USA).

### Tumor treatment regimens

Mice were developed tumor subcutaneously with 5 × 10^4^ TC-1 cells or 4T1-Luc2 cells. All animal studies were in accordance with the National Institutes of Health guide for the care and use of Laboratory animals (NIH Publication No.85-23, revised 1996), and were institutionally approved by the Institutional Animal Care and Use Committee, Institute of Biophysics, Chinese Academy of Sciences (SYXK2017-07). When primary tumor volume was 120–200 mm^3^ for TC-1 model and 80–150 mm^3^ for 4T1-Luc2 model, mice underwent radiation treatment. Single dose of 12 Gy (1.8 Gy/min) RT (γ-ray from ^60^Co source, College of Chemistry and Molecular Engineering, Peking University, China) was delivered to tumor. Dosimetry was determined with thermoluminescent dosimeters^54^. For vaccine plus radiation combination therapy, TC-1 tumor-bearing mice received E7 vaccine (5 µg/mouse, sc.) on day 11 after tumor implantation and then received a single dose of radiation, followed by receiving another 3 consecutive doses of E7 vaccine. For 4T1-Luc2 model, Luc2 vaccine was given (6 µg per mouse, sc.) 3 days before radiation and then administered a single radiation dose, followed by administering additional 3 consecutive doses of Luc2 vaccine. For radiation plus vaccine therapy, tumor was given a single radiation dose followed by administration of 4 consecutive doses of E7 vaccine.

In vivo depletion of CD25^+^ (IL-2Ra) and CD8^+^ T cells was achieved by intraperitoneal injection of anti-mouse CD25 (Clone: PC-61.5.3) or anti-mouse CD8 (Clone: 2.43) antibodies (BioXcell) at a dose of 200 µg per mouse, starting the therapy on the same day of vaccine administration. Tumor volume was measured twice a week and calculated as length × width × height.

The lung metastases were determined by bioluminescence using the IVIS Spectrum In Vivo Imaging System (Xenogen, Caliper Life Science, PerkinElmer, Hopkinton, MA, USA) as mentioned earlier^55^.

### Flow cytometry analysis

Single-cell suspensions were prepared as mentioned previously^55^. Samples were stained (20–30 min) with the following antibodies: anti-CD45, anti-CD3, anti-CD4, anti-CD8a, anti-CD25, anti-CD11b, anti-Gr-1, anti-MHC I, anti-CXCR3, and anti-CCR5 antibodies. For intracellular staining, cells were fixed, permeabilized overnight at 4 °C (Fixation/Permeabilization Concentrate and Diluent kit, eBioscience), and subsequently stained using anti-IFN-γ or anti-Foxp3 and analyzed by flow cytometry (BD FACSCallibur). All antibodies were collected from eBioscience (San Diego, CA, USA) or BioLegend (USA).

### Cytotoxic T lymphocyte assay (CTL) in vitro

In order to evaluate the degree of CTL level by E7 vaccine or Luc2 vaccine, naïve mice were administered subcutaneous injection of E7 vaccine or Luc2 vaccine at 0 and 7 days. Both spleens and inguinal lymph nodes were collected from immunized mice on day 14 and single cell suspensions were prepared. The cells were seeded (10^6^ cells/100 μL) in U-shaped 96-well plates and incubated in CO_2_ cell incubator for 24 h, followed by stimulation with HPV16 E7_49-57_ peptides or Luc2 peptides (5 μg/mL) in the presence of Brefeldin A (5 μg/mL) (eBioscience) for 6 h and finally, cells were stained with anti-CD8 antibody, fixed, permeabilized, and stained with anti-IFN-γ antibody. The percentages of CTL (CD8^+^IFN-γ^+^ cells) were detected using flow cytometry analysis.

### RNA isolation and quantitative real-time PCR

Isolation of whole RNA from tumor tissue was carried out using TRIzol reagent (Invitrogen). cDNA templates were obtained by TransScript One-Step gDNA Removal and cDNA Synthesis SuperMix (Transgen) following the manufacturer’s guidelines. TransSmart^TM^ Green qPCRSuperMix UDG (Transgen) on ABI 7300 was used for performing the quantitative real-time PCR (qRT-PCR).

### Immunofluorescence staining

Formalin fixed (10%), paraffin-embedded sections were prepared for immunofluorescence staining to detect CD8a. After dewaxing and dehydration, sections were adsorbed in 1% tween-20, and heat-induced antigen retrieval was conducted by Target Retrieval Solution (Dako, Santa Clara, CA) for 45 min. Before primary antibody treatment, tumor slides were washed with PBST and endogenous peroxidise, followed by blocking using Dual Endogenous Enzyme Block (Dako) and then sections were stained with 5 µg/mL anti-mouse CD8a monoclonal antibody (4SM15, Invitrogen) at 4 °C overnight. The primary antibody detection was performed by a fluorescence-conjugated secondary antibody for 1 h, the nuclei were stained with 300 nM of DAPI solution for 10 min, and then fluorescent detection was conducted using an upright fluorescence microscopy. Positive staining cells were counted and analyzed using Image-Pro Plus software.

### CD8^+^ T cells migration detection

T cells migration assay was carried out with slight modification^33^. Briefly, cells (1 × 10^6^ cells/mL, 100 μL) were added into top well and allowed to migrate at 37 °C for 24 h. The lower chamber was filled with 1 mL supernatant of radiation treatment (X-ray: 8 Gy) or non-treatment TC-1 cells (2 × 10^4^ cells/mL). After incubation, migrated cells were collected and stained with anti-CD3 and anti-CD8 antibodies and cells were counted by FACS at a constant flow rate for 90 s.

### Protein isolation and chemokine level detection by flow cytometry

Tumor tissues were made into powder, followed by adding protein extraction buffer (Tris-HCl, NP-40, NaCl, EDTA, NaN3, and PMSF at pH 7.5) and protease inhibitors (DTT, leupeptin and aprotinin) and then the lysate was centrifuged at 27,000*g* for 20 min and the protein was collected from the supernatant.

The expression level of chemokine in protein was analyzed according to manufacturer’s instructions (Mouse Proinflammatory Chemokine Panel with Filter Plate, Multi-Analyte Flow Assay Kit, BioLegend). Briefly, diluted standards or samples (25 μL) were taken and then added 25 μL of assay buffer, 25 μL of mixed beads, and 25 μL of detection antibodies followed by shaking at 1000 RPM for 2 h. Finally, added 25 μL of SA-PE and removed the supernatant followed by washing, then resuspended and measured the chemokines.

### In vitro analysis of CXCR3, CCR5, and chemokine expression by co-culture of T cells and tumor cells

To determine the CXCR3 and CCR5 expression level by E7 vaccine treatment, naïve mice were subcutaneously injected two doses of vaccine at day 0 and 7. Briefly, cells were seeded (10^6^ cells/100 μL) in 96-well plate with U bottom and incubated for 24 h, followed by re-stimulation with E7 short peptides in the presence of Brefeldin A (5 μg/mL) (eBioscience) for 6 h. Finally, cells were stained with anti-mouse CD8-FITC and anti-CXCR3-PE or anti-mouse CD8-PE and anti-CCR5-Alexa Fluor 488 antibodies, fixed, permeabilized, and stained with anti-mouse IFN-γ-APC antibody.

Irradiated TC-1 cells (X-ray: 8 Gy) were seeded (2 × 10^4^ cells/mL) in 24-well plates with or without stimulation of IFN-γ (50 ng/mL) and incubated for 24 h prior to adding the vaccinated T cells. Supernatant T cells and attached TC-1 cells were collected 48 h after co-culture and performed chemokine expression evaluation using flow cytometry analysis (Multi-Analyte Flow Assay Kit, BioLegend).

### Statistics and reproducibility

Data were presented as mean ± SD. Analysis of results containing two groups was carried out using the Student’s *t*-test, assuming unequal variance. The survival curves were analyzed using log-rank (Mantel–Cox test) analysis. Protein-level chemokines were analyzed by LEGENDplex v8.0 software. In all the tests, *P* < 0.05 was considered to be statistically significant.

### Reporting summary

Further information on research design is available in the Nature Research Reporting Summary linked to this article.

## Supplementary information

Supplementary Information

Description of Additional Supplementary Files

Supplementary Data 1

## Data Availability

All data generated or analyzed during the current study are included in this published article (and its supplementary information files) and all relevant data are available from the corresponding authors upon request. Source data underlying the graphs can be found in Supplementary Data 1 and the microscopy images of Fig. 4d are shown in Supplementary Fig. 7.
