# Supplementary material for: Optimal combination treatment regimens of vaccine and radiotherapy augment tumor-bearing host immunity
Source: Commun Biol. 2021 Jan 19;4:78. doi: 10.1038/s42003-020-01598-6 (PMC7815836; doi:10.1038/s42003-020-01598-6)
Supplement: Supplementary file 1 — Supplementary Information [file 42003_2020_1598_MOESM1_ESM.pdf]

Optimal combination treatment regimens of vaccine and radiotherapy augment tumor-bearing host immunity

Fayun Zhang[1*#], Zifeng Zheng[1,2#], Apurba Kumar Barman[3#], Zihao Wang[1], Luyao Wang[1], Wenfeng Zeng[1], Luoyang Wang[4], Yan Qin[1], Asmita Pandey[1], Chunling Zhang[1], Wei Liang[1,2*]

[1]Protein & Peptide Pharmaceutical Laboratory, Institute of Biophysics, Chinese Academy of Sciences, Beijing 100101, PR China

[2]University of Chinese Academy of Sciences, Beijing 100049, PR China

[3]Pharmacology Laboratory, Department of Pharmacy, School of Life Science and Health, Ranada Prasad Shaha University, Naryanganj-1400, Bangladesh

[4]Department of chemical engineering, Tsinghua University, Beijing 100084, PR China

Corresponding Authors:

[*]Fayun Zhang and Wei Liang, Protein & Peptide Pharmaceutical Laboratory, Institute of Biophysics, Chinese Academy of Sciences, Beijing 100101, China. Phone: 8610-64889861. E-mail: fyzhang@ibp.ac.cn(Fayun Zhang) and weixx@ibp.ac.cn (Wei Liang)

[#]These authors contributed equally：Fayun Zhang, Zifeng Zheng and Apurba Kumar Barman.

Supplementary Figures

Supplementary Fig. 1

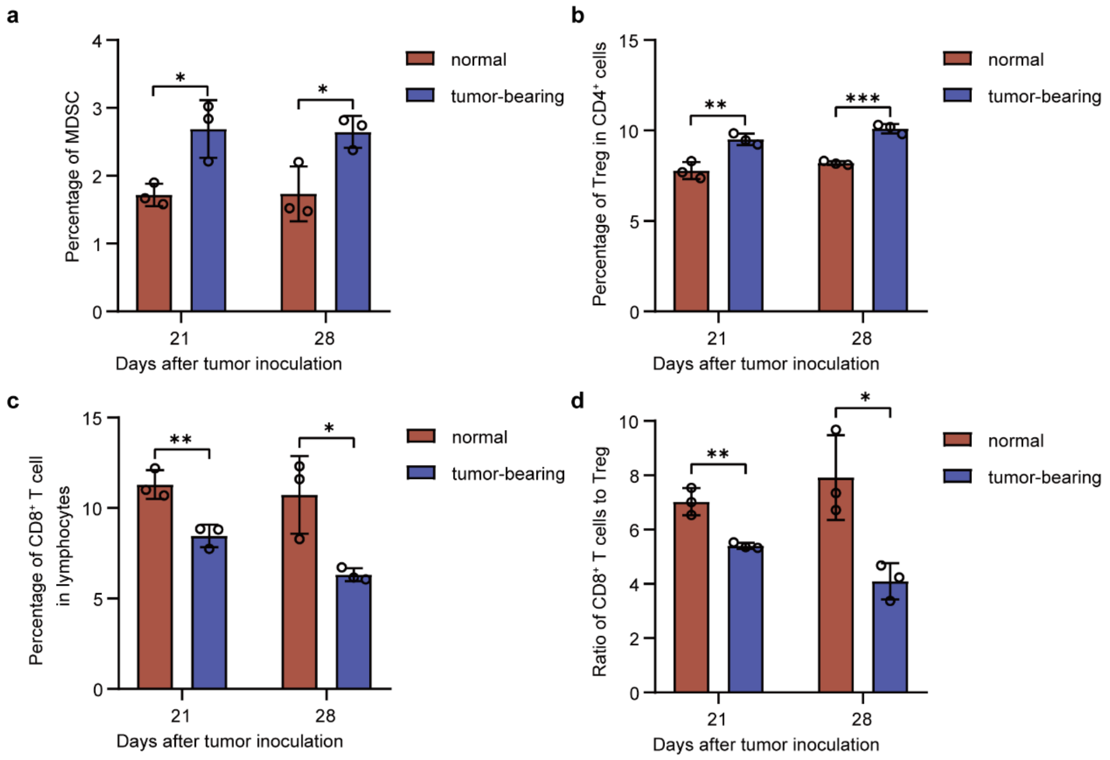

Supplementary Figure 1. Host immunosuppression was associated with tumor progressing. Local tumors in C57BL/6J mice were established using TC-1 cells and then 21, and 28 days after tumor cells inoculation, the spleens were collected. (**a-d**) Flow cytometry analysis. (**a**) Percentage of MDSCs in spleens. (**b**) Percentage of Tregs in CD4+ cells in spleens. (**c**) Percentage of CD8+ T cells of lymphocytes in spleens. (**d**) Ratio of CD8+ T cells to Tregs in spleens. Data are presented as mean ± SD; n = 3 mice/group. Experiments were repeated three times and two-tailed t-test was used for comparisons of biological replicates. *P <0.05, ***P <0.01, ***P <0.001.

Supplementary Fig. 2

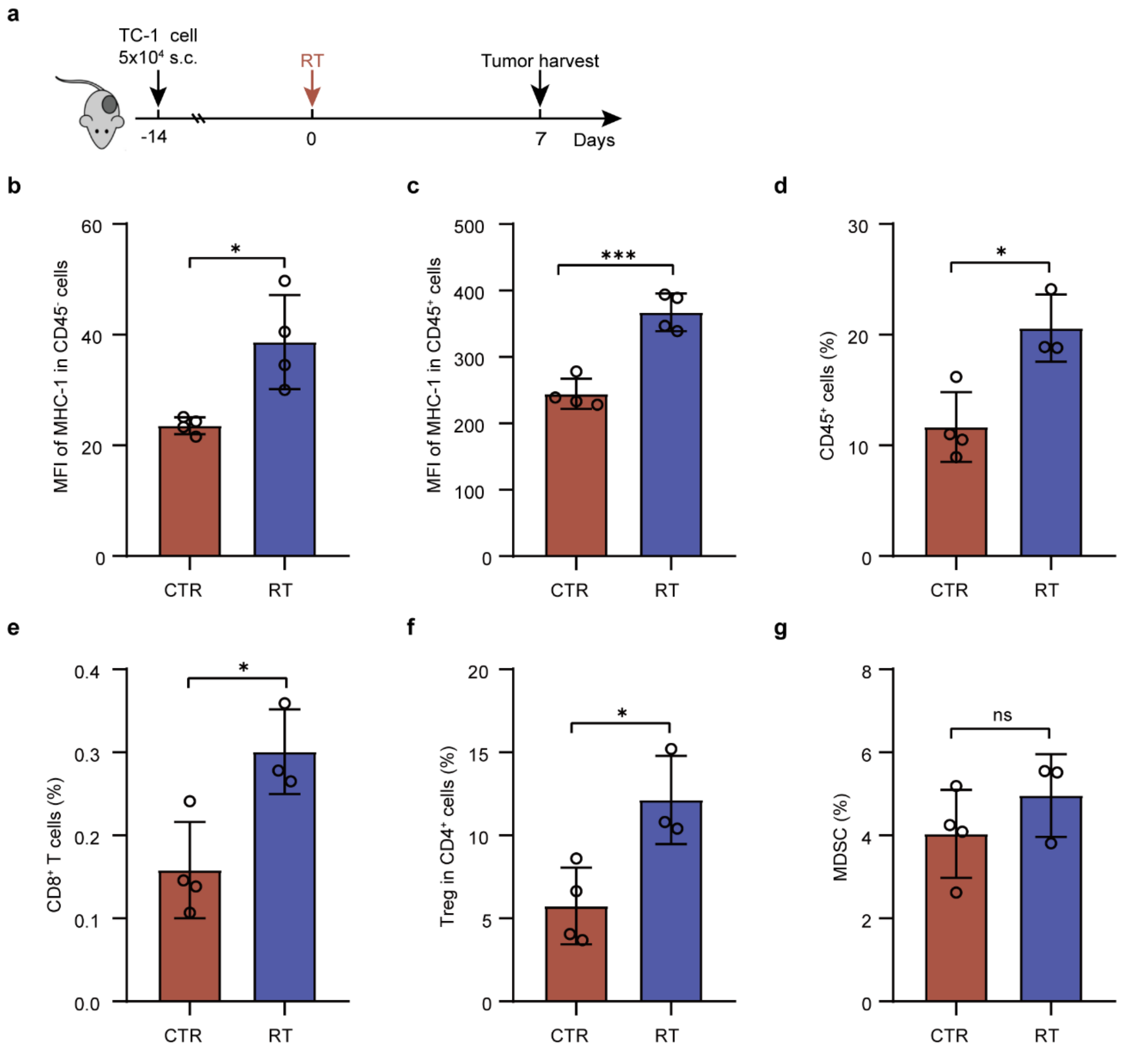

Supplementary Figure 2. Radiation induced leukocytes accumulation and Tregs increase in TME. (**a**) Experimental procedure. The local tumor was established using 5×10^4 TC-1 cells by subcutaneous (s.c.) injection beside the forth fat-pad of wild-type C57BL/6J mouse. The tumor-bearing mice received radiation at a dose of 12 Gy on day 14 after tumor cells injection and then 7 days after RT, tumor tissues were harvested. (**b-f**) Flow cytometry analysis. (**b**) MFI (Mean Fluorescence Intensity) of MHC-1$^+$ cells in CD45$^-$ cells in tumors. (**c**) MFI of MHC-1$^+$ cells in CD45$^+$ cells in tumors. (**d**) Percentage of CD45$^+$ cells in tumors. (**e**) Percentage of CD8$^+$ T cells in CD45$^+$ cells. (**f**) Percentage of Tregs in CD4$^+$ cells. (**g**) Percentage of MDSCs in CD45+ cells in tumors. Data are presented as mean ± SD; n=3 to 4 mice/group. Experiments were repeated two times and two-tailed *t*-test was used for comparisons of biological replicates. *P <0.05, ***P <0.001.

Supplementary Fig. 3

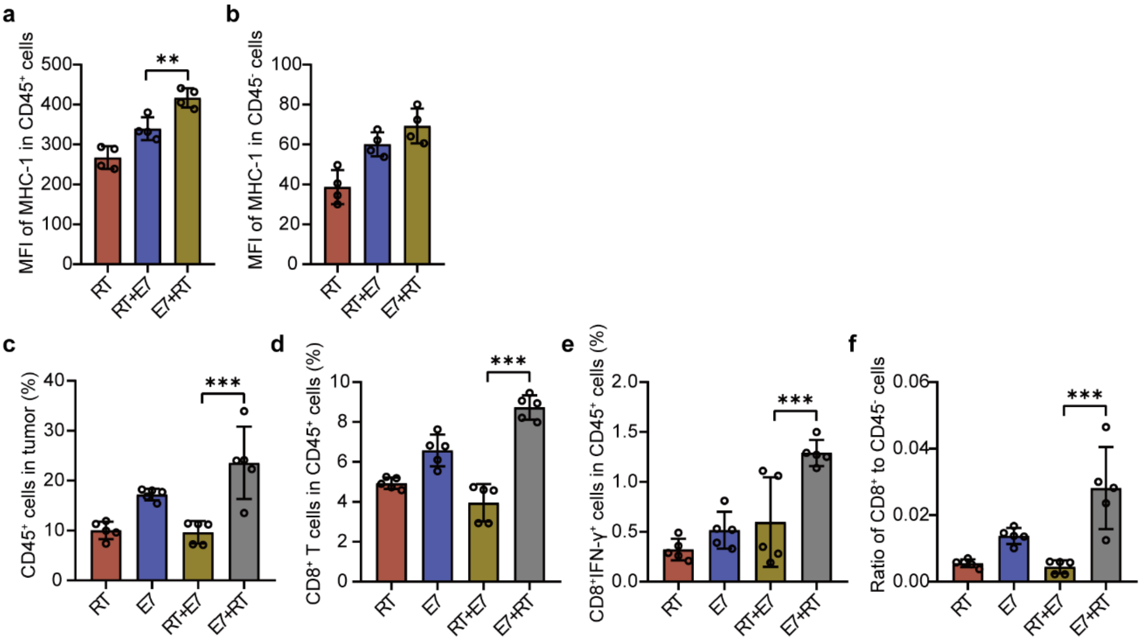

Supplementary Figure 3. Vaccination prior to radiation therapy induced more CD8$^+$IFN-γ$^+$ cells infiltrating into tumors. TC-1 tumors were established and treated by E7 vaccine plus RT, RT alone (14 days after tumor inoculation), and RT plus E7 vaccine, respectively. The tumors were collected 14 days after RT. Flow cytometry analyses were then performed with TC-1 tumors. (**a**) The MFI of MHC-1$^+$ cells in CD45$^+$ cells, and (**b**) the MFI of MHC-1$^+$ cells in CD45$^-$ cells in the tumors. n= 4 mice/group. (**c**)The percentage of CD45$^+$ cells in the tumors determined by FACS. (**d**) The percentage of CD8$^+$ cells in CD45$^+$ cells in the tumors. (**e**) The percentage of CD8$^+$IFN-r$^+$ cells in CD45$^+$ cells in the tumors analyzed by flow cytometry. (**f**) The ratio of CD8$^+$/CD45$^-$ cells in tumors analyzed using flow cytometry. n= 5 mice/group. Data were presented as mean ± SD; Experiments were repeated two times and two-tailed *t*-test was used for comparisons of biological replicates. **P <0.01, ***P <0.001.

Supplementary Fig.4

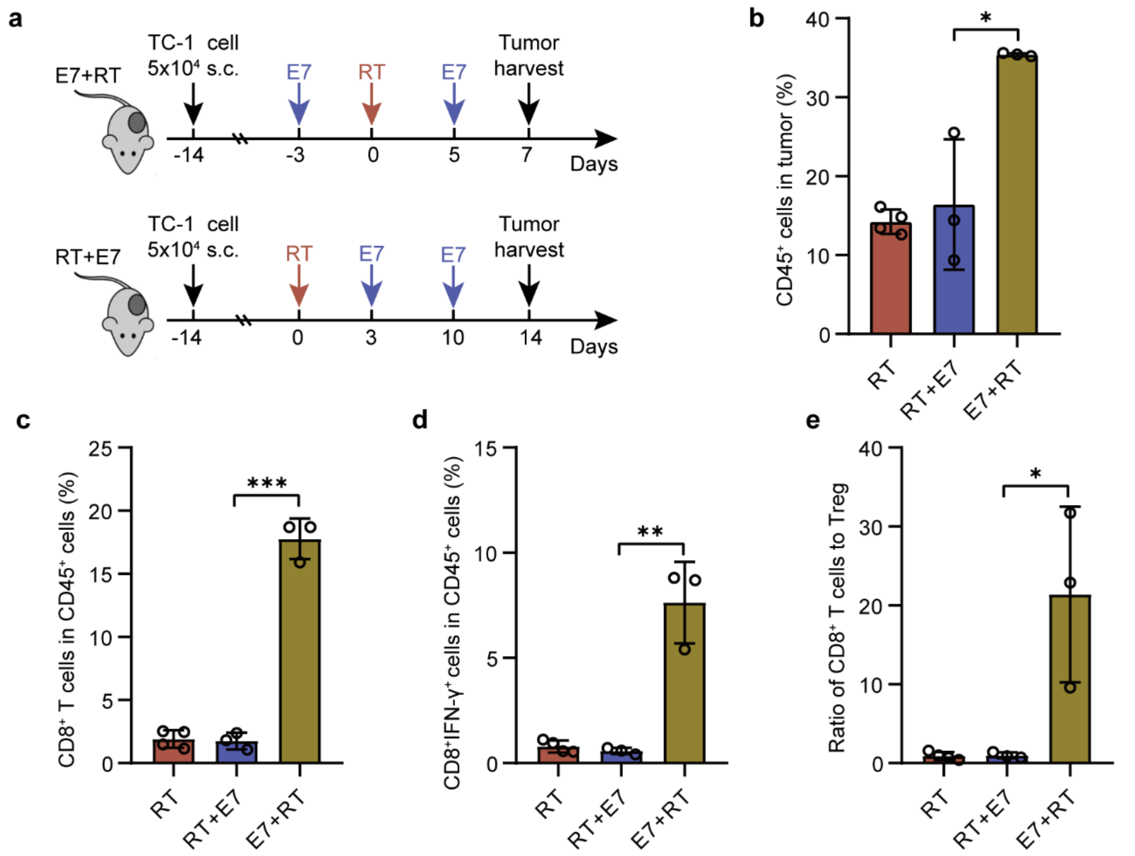

Supplementary Figure 4. (**a**) Experimental procedure of TC-1 tumor model and treatments. The tumor-bearing mice received radiation at a dose of 12 Gy on day 14 after tumor cells injection. After 2 doses of vaccine, tumor tissues were harvested. (**b-e**) Flow cytometry analyses. (**b**) Percentage of $CD45^+$ cells in tumors. (**c**) Percentage of $CD8^+$ T cells of $CD45^+$ cells in tumors.(**d**) Percentage of $CD8^+$ IFN-r^+ cells of $CD45^+$ cells in tumors. (**e**) Ratio of $CD8^+$ T cells to Tregs in tumors. Data are presented as mean ± SD; n=4 mice for Control group and 3 mice for each other group. Experiments were repeated two times and two-tailed *t*-test was used for comparisons of biological replicates. $^*P < 0.05$, $^{**}P < 0.01$, $^{***}P < 0.001$.

Supplemental Fig.5

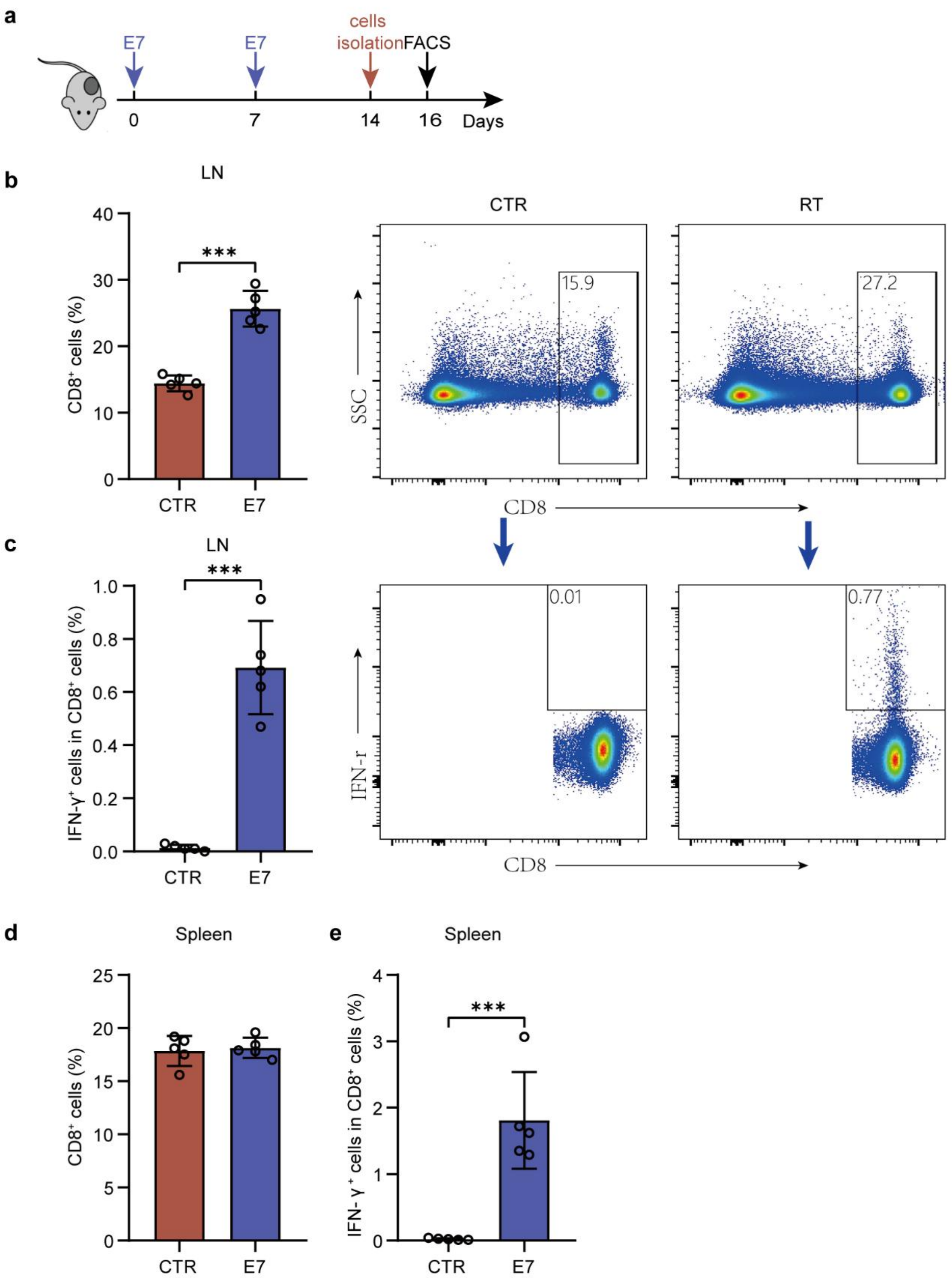

Supplementary Figure 5. The micelle-based E7 vaccine induced a higher level of antigen-specific potent CTLs. (**a**) Experimental procedure. C57BL/6J mice were inoculated (s.c.) with two doses of E7 vaccines containing 5μg E7 and 2.5μg MPLA

per mouse. After 7 days of last vaccination, lymphocytes were isolated from the inguinal lymph nodes and spleens. The cells were seeded and stimulated with $E7_{49-57}$ peptide (5μg/ml) for 6h. (**b-e**) Flow cytometry analysis. (**b**) The percentage of $CD8^+$ cells in lymphocytes (**Left**) and representative flow cytometry plot of $CD8^+$ T cells (**Right**) of lymph nodes. (**c**) The percentage of $CD8^+IFN\text{-}\gamma^+$ cells in $CD8^+$ lymphocytes (**Left**) and representative flow cytometry plot of $CD8^+IFN\gamma^+$ cells (**Right**) of lymph nodes. (**d**) The percentage of $CD8^+$ T cells in lymphocytes of spleens. (**e**) Percentage of $CD8^+IFN\text{-}\gamma^+$ cells in $CD8^+$ lymphocytes of spleens. Results were presented as mean ± SD; n = 5 mice per group. Experiments were repeated three times and two-tailed *t*-test was used for comparisons of biological replicates. ***P <0.001.

Supplementary Fig. 6

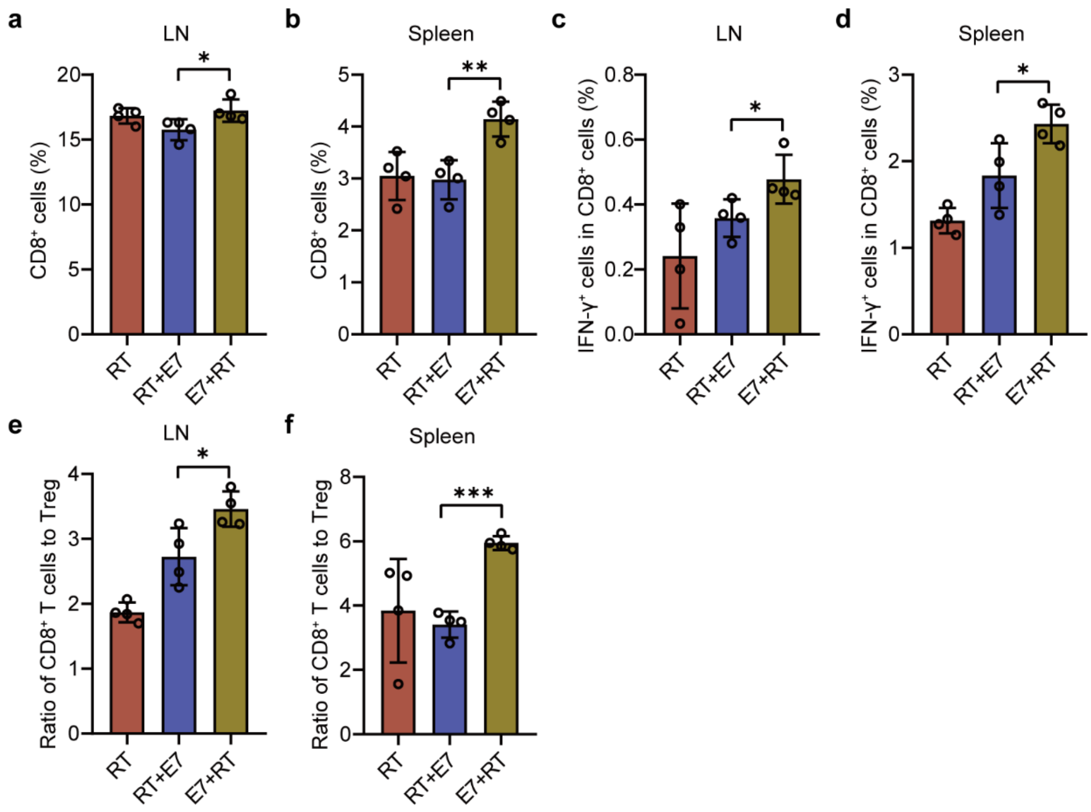

Supplementary Figure 6. Tumors treated by the combinations of E7 vaccines before or after radiation altered the percentage of CD8$^+$IFNr$^+$ and the ratio of CD8$^+$/Treg cells in lymph nodes and spleens. The percentage of CD8$^+$ T cells and IFNr$^+$ cells in CD8$^+$ T cells, the ratio of CD8$^+$ T cells to Tregs in the lymphocytes of spleens and lymph nodes after mice received combined radiotherapy for 14 days. C57BL/6 mice were s.c injected with 5X10^4 TC-1 tumor cells and the experimental procedures were the same to that described in Figure 3a. (**a-b**) The percentage of CD8$^+$ cells in lymphocytes of lymph node cells (**a**) and in spleen lymphocytes (**b**). (**c-d**) The percentage of IFNγ$^+$ cells in CD8$^+$ cells in lymph node cells (**c**) and in spleen lymphocytes (**d**). (**e-f**) The ratio of CD8$^+$/Tregs in lymph node cells (**e**) and in spleen lymphocytes (**f**) analyzed using flow cytometry. Experiments were repeated two times and two-tailed *t*-test was used for comparisons of biological replicates. Data are presented as mean ± SD; n= 4 mice/group. *P <0.05, **P <0.01, ***P <0.001.

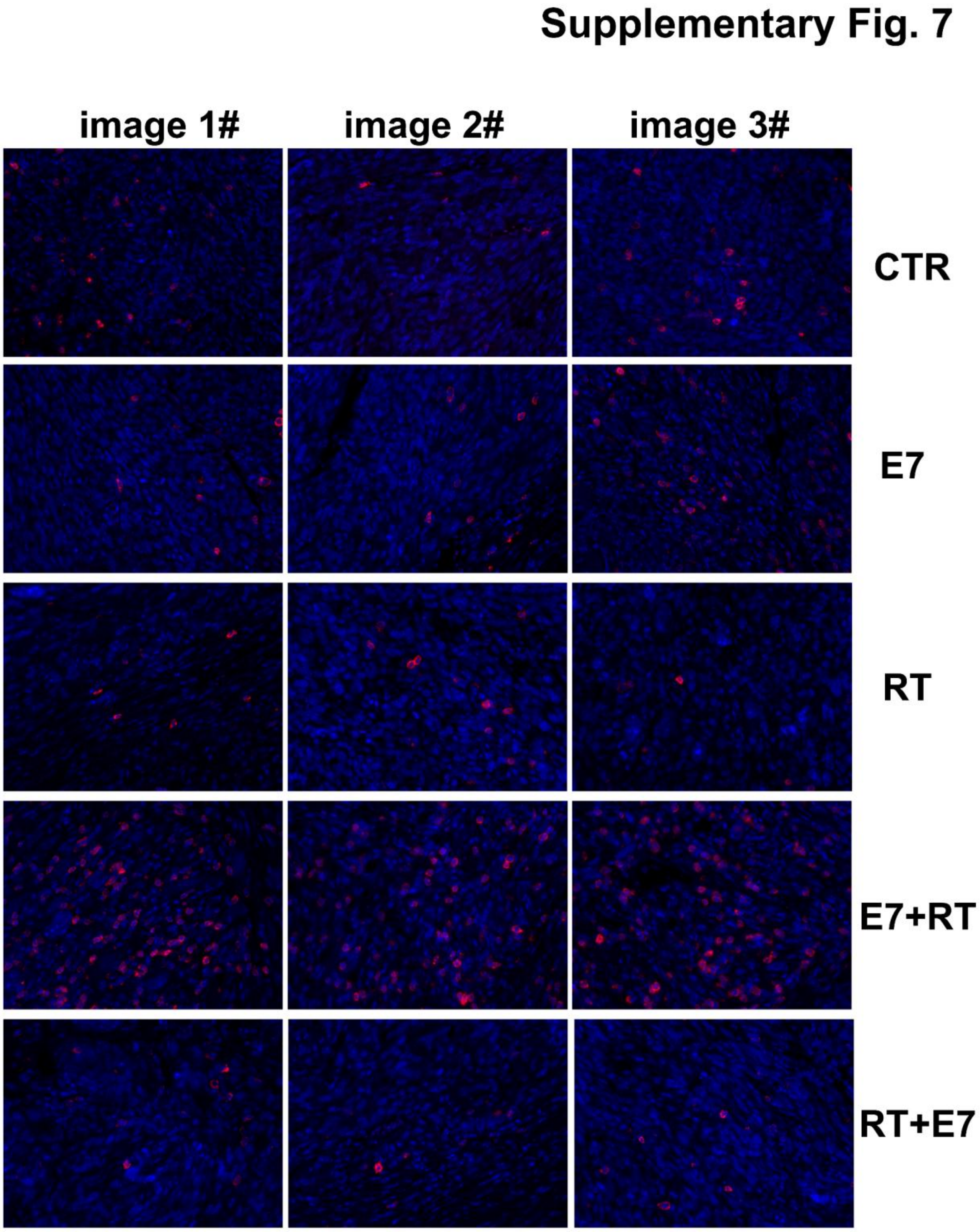

Supplementary Figure 7. Representative immunofluorescent images taken from tumors in different treatment groups. Slides were incubated with anti-CD8 primary antibodies overnight in 4˚C and then with corresponding secondary antibodies (RED), then stained nucleus with DAPI (BLUE). The pictures were merged by image software. 3 tumors each group were presented as 3 slide images.

Supplementary Fig. 8

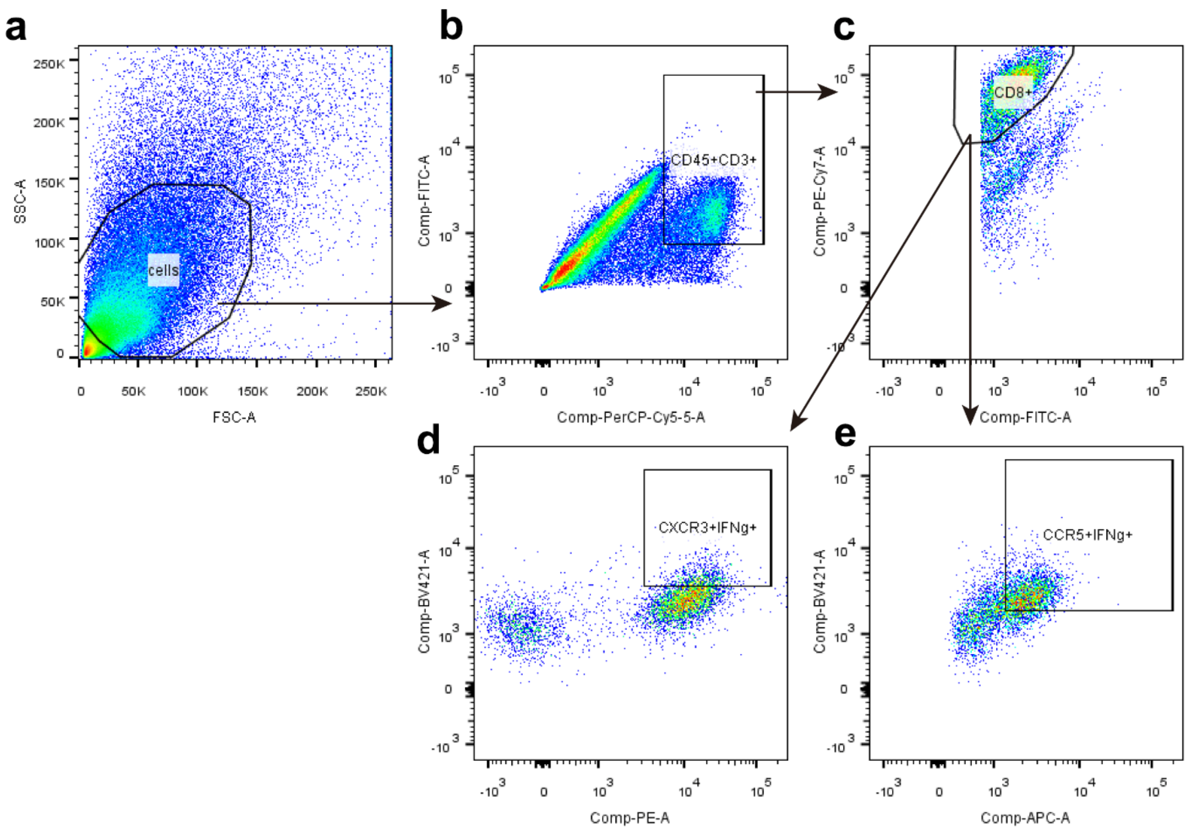

Supplementary Figure 8. FACS gating strategy for the sorting of CD8$^+$CXCR3$^+$IFN-γ$^+$ and CD8$^+$CCR5$^+$IFN-γ$^+$ cells from tumor tissue suspension. (**a)** First plot gating for live cells. (**b)** Second plot for double positive expression of CD45$^+$CD3$^+$ cells. (**c**) The gating plot for CD8$^+$ cells in CD45$^+$CD3$^+$ cells. (**d-e**) The gating plot for CXCR3$^+$IFN-γ$^+$ (**d**) and CCR5$^+$IFN-γ$^+$ cells in CD8$^+$ cells (**e**).

Supplementary Fig. 9

a

	Epitope (sequence)
Luc2$_{32}$	R Y A L V P G T I
Luc2$_{160}$	G F Q S M Y T F V
Luc2$_{293}$	S F F A K S T L I
Luc2$_{446}$	G Y Q V A P A E L

b

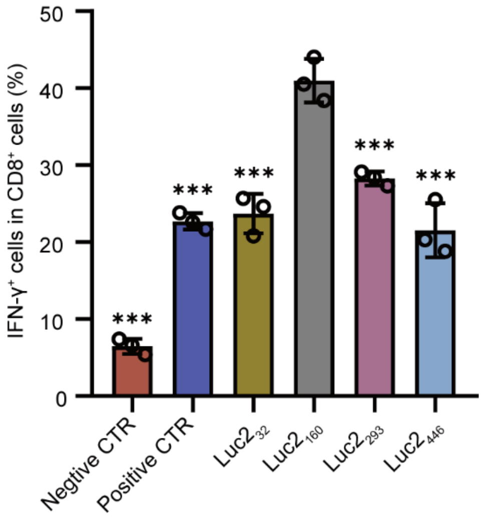

Supplementary Figure 9. Antigen-specific CTL levels induced by the micelle-based Luc2 vaccines with different epitopes. BALB/c mice received (s.c.) two doses of different Luc2 epitopes vaccine formulations containing 6μg of Luc2 peptides and 2.5μg of MPLA per mouse. After 7 days of the last vaccination, lymphatic cells were isolated from the inguinal lymph nodes, followed by the cells seeding and stimulation with Luc2 short peptides for 6h. (**a**) Sequences of Luc2 epitopes. (**b**) Percentage of $CD8^{+}IFN\text{-}\gamma^{+}$ cells were measured by flow cytometry. Results are presented as mean ± SD; n=3 mice per group. Experiments were repeated two times and two-tailed *t*-test was used for comparisons of biological replicates. ***P <0.001.
