# Supplementary material for: Optimal combination treatment regimens of vaccine and radiotherapy augment tumor-bearing host immunity
Source: Commun Biol. 2021 Jan 19;4:78. doi: 10.1038/s42003-020-01598-6 (PMC7815836; doi:10.1038/s42003-020-01598-6)
Supplement: Supplementary file 2 — Description of Additional Supplementary Files [file 42003_2020_1598_MOESM2_ESM.pdf]

Description of Additional Supplementary File

File Name: Supplementary Data 1

Description: Source data for all graphs in the main figures and supplementary figures.
